# Delivery of Chemotherapy Agents and Nucleic Acids with pH-Dependent Nanoparticles

**DOI:** 10.3390/pharmaceutics15051482

**Published:** 2023-05-12

**Authors:** Qixin Leng, Zuha Imtiyaz, Martin C. Woodle, A. James Mixson

**Affiliations:** 1 Department of Pathology, University Maryland School of Medicine, University of Maryland, 10 S. Pine St., Baltimore, MD 21201, USAzimtiyaz@som.umaryland.edu (Z.I.); 2 Norwood Bio, 1805 College Avenue, Alamogordo, NM 88310, USA; mwoodle@gmail.com

**Keywords:** polymers, pH-sensitive, nucleic acids, siRNA, chemotherapy, nanoparticles, carriers, tumor pH

## Abstract

With less than one percent of systemically injected nanoparticles accumulating in tumors, several novel approaches have been spurred to direct and release the therapy in or near tumors. One such approach depends on the acidic pH of the extracellular matrix and endosomes of the tumor. With an average pH of 6.8, the extracellular tumor matrix provides a gradient for pH-responsive particles to accumulate, enabling greater specificity. Upon uptake by tumor cells, nanoparticles are further exposed to lower pHs, reaching a pH of 5 in late endosomes. Based on these two acidic environments in the tumor, various pH-dependent targeting strategies have been employed to release chemotherapy or the combination of chemotherapy and nucleic acids from macromolecules such as the keratin protein or polymeric nanoparticles. We will review these release strategies, including pH-sensitive linkages between the carrier and hydrophobic chemotherapy agent, the protonation and disruption of polymeric nanoparticles, an amalgam of these first two approaches, and the release of polymers shielding drug-loaded nanoparticles. While several pH-sensitive strategies have demonstrated marked antitumor efficacy in preclinical trials, many studies are early in their development with several obstacles that may limit their clinical use.

## 1. Introduction

Chemotherapeutic agents frequently are cytotoxic drugs, which, in most instances, have different mechanisms of action in various cell cycle phases. The inability of chemotherapy to differentiate clearly between malignant and normal cells leads to adverse events and toxicities. Moreover, most chemotherapeutic agents are administered in high dosages intravenously to obtain therapeutic levels in the tumor. Thus, high drug dosages have a double-edged sword in patients. While these dosages enable the agents to achieve therapeutic effects, they also cause significant untoward side effects for patients [1]. The inability to reduce the chemotherapy levels in normal tissues limits antitumor efficacy [2,3]. Furthermore, given the vasculature’s complexity and typically short half-life in the blood, limited amounts of the drug reach the tumor site. As a result, enhancing delivery and increasing tissue specificity are critical issues in developing improved efficacy for chemotherapeutic agents.

One approach has been to incorporate these agents into nanoparticles to minimize toxicity and increase the efficacy of chemotherapy agents. Nanoparticles greater than 8–10 nm accumulate in tumor tissues by enhanced permeability and retention (EPR) [4]. The EPR mechanism combines leaky tumor blood vessels and reduced numbers of tumor lymphatic vessels resulting in the influx of nanoparticles and decreased drainage of nanoparticles. To take advantage of the passive EPR effect, various drug nanocarriers, including lipid- and polymer-based carriers, have been used in pre-clinical and clinical tumor studies [5,6,7,8,9,10,11]. The FDA-approved Doxil^®^, a liposomal preparation of doxorubicin (Dox), has successfully used this approach. Compared to those administered free Dox, patients given Doxil^®^ have less cardiotoxicity, nausea, and myelosuppression [12,13]. Fewer adverse side effects are likely due to the accumulation of nanoparticles (i.e., Doxil^®^) in the tumor (and avoidance of other tissues). Still, the survival of cancer patients treated with Doxil^®^ has not been improved compared to free Dox, except in a subset of patients with cardiomyopathy [14]. More recently, Onivyde^®^, a liposomal irinotecan product, was approved in 2015 as a second-line therapy for pancreatic cancer, and is undergoing evaluation for naïve-treated metastatic cancer patients [15]. Unlike Dox and irinotecan, other chemotherapeutic agents, such as cisplatin or paclitaxel (PTX), have not been loaded efficiently into liposomes [16]. Nonetheless, there are non-liposomal polymeric nanoparticles with high loading capacity for PTX and prolonged half-lives in the bloodstream, which have demonstrated marked antitumor efficacy [17,18]. Although several clinical trials are evaluating the efficacy of polymeric-chemotherapy agents, none of these have been approved for clinical use by regulatory agencies [10,19]. Alternatively, the Abraxane^®^ nanoparticle, an albumin platform for PTX, has effectively treated advanced breast, pancreatic, and lung cancers. However, we are not aware of any pH-dependent strategies that are being evaluated in clinical trials for the treatment of cancer.

By increasing the half-life of polymeric and liposomal carriers through pegylation, the accumulation of the nanoparticles in the tumor may be more significant based on the EPR effect. One drawback to pegylated products is that a small percentage of patients have an immunological reaction ranging from reduced efficacy to anaphylaxis [20]. Tumor accumulation of the nanoparticles may also be increased by several factors, including the charge, size, and shape of the particle [21]. In addition, active tumor targeting by the inclusion of tumor-specific ligands on the nanoparticle’s surface can enhance tumor accumulation and anti-tumor efficacy (at least in pre-clinical trials) [22]. Despite modifications of nanoparticles, a major obstacle is their low tumor accumulation. One meta-analysis of 117 studies reported a median of 0.7 percent of the injected dose reached the tumor [23]. To improve tumor accumulation of nanoparticles, several novel mechanisms or approaches are now being explored to direct and release the therapy in or near tumors [24,25]. A promising approach that applies to passive and active targeting has been stimuli-based therapies that release agents in the peritumoral region through changes in redox potential, temperature, enzymatic reaction, pH, or external stimuli such as heat and light.

This review will focus on pH-dependent targeting of chemotherapeutic agents, an area of immense interest for tumor therapy in recent years. Four types of pH-dependent targeting of the tumor will be explored, including pH-sensitive linkages, protonation and disruption of the nanoparticles, a combination of these first two approaches, and finally, the release of coating polymers from the drug-loaded nanoparticles. The first approach depends on the breakage of the pH-sensitive bond, resulting in the release of the chemotherapy agent from the carrier and nanoparticle under acidic conditions. The second approach examines targeting tumors and disrupting nanoparticles due to a lower pH in the tumor matrix and endosomes. Protonation of these polymeric nanocarriers leads to their disassembly due to charge–charge repulsion in an acidic environment. Like other polymeric nanoparticles, pH-sensitive particles comprise block-copolymers with hydrophilic and hydrophobic domains that self-assemble into structures with a hydrophobic core and hydrophilic shell. The third approach is an overlap of the first two approaches. That is, both pH-sensitive linkages and disruption of nanoparticles have significant roles in the release of chemotherapy drugs and nucleic acids. Finally, the fourth approach will investigate the pH-dependent coating of nanoparticles with the release of drugs in mildly acidic environments. To varying degrees, the four strategies overlap and are contingent on the acidic extracellular pH (pHe) of the tumor and the well-established acidic pH of the tumor cell endosomes.

## 2. Acidic Tumor Environments

**Figure 1:**
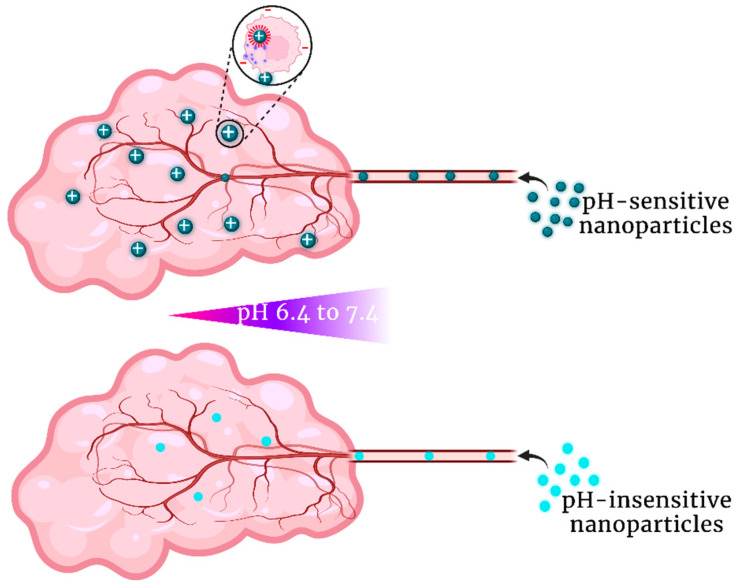
Accumulation of pH-sensitive nanoparticles based on pH gradient between the blood and the tumor cell membrane. As the nanoparticles enter the acidic tumor matrix, the particles become more positively charged, enabling their interaction with tumor cell membranes. With non-pH-responsive nanoparticles, tumor accumulation occurs to a lesser degree [27,28].

The nanoparticles and the drugs they carry will be exposed to different pH neighborhoods as they traverse blood vessels to their tumor cell targets. Two of these are acidic—the extracellular tumor matrix and endosomes within tumor cells—whereas the cytosol of the tumor cells is relatively alkaline (compared to normal cells). These environments likely interact to regulate their pH and enhance the aggressiveness of the tumor [26]. Moreover, these acidic and alkaline environments affect the drug’s efficacy against the tumor in at least three ways. First, the pH gradient between the blood and the tumor cell membrane may affect the accumulation of pH-sensitive nanoparticles (Figure 1) [27]. Second, the more acidic endosomes likely have a greater role in disrupting the particles and releasing the drug than the less acidic tumor matrix. Third, the pH gradient between the acidic environment and the more alkaline tumor cytosol may affect the distribution and activity of the released drug in the tumor cell. After examining the acidic tumor environments in which nanoparticles are exposed, we will examine some recent advances in pH-dependent nanoparticles.

### 2.1. The Extracellular pH (pHe) of Tumors

**Figure 2:**
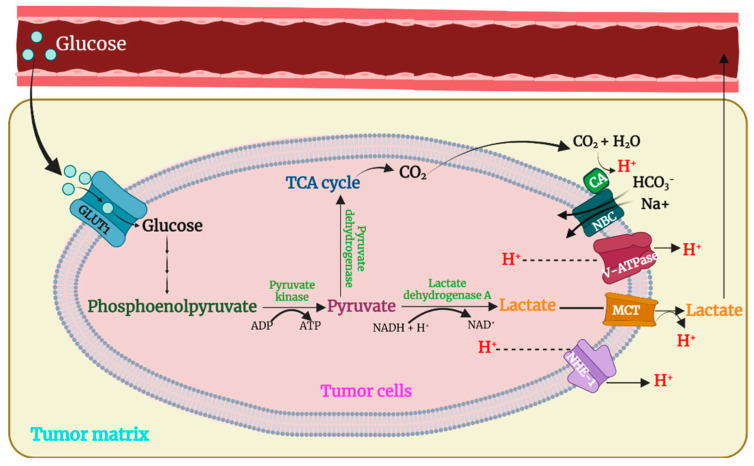
Metabolic and proton pump alterations in tumors leading to an acidic extracellular pH. Increased glucose import (e.g., Glut), aerobic and anaerobic glycolysis, and increased proton pumps (MCT, NHE-1, and V-ATPase) reduce extracellular pH in solid tumors. Because of proton pumps and bicarbonate transporters (NBC), the intracellular pH of tumor cells increases, resulting in a pH reversal [31,32,33]. MCT, monocarboxylate transporter, NHE-1, sodium hydrogen exchange, V-ATPase, vacuolar ATPase transporter, NBC, sodium bicarbonate transporter, and CA, carbonic anhydrase.

Approximately a century ago, Otto Warburg determined that cancer cells preferentially ferment glucose to lactate even when sufficient oxygen supports oxidative phosphorylation [29,30]. Although increased glycolytic activity with the production of lactic acid and its export was initially thought to be the primary reason for the lower extracellular pH in solid tumors [30], several additional mechanisms have significant roles (Figure 2). These mechanisms include an increase in membrane proton pumps, bicarbonate transport exchanges, sodium-hydrogen exchanges, carbonic anhydrase activity, and HIF-1α levels (See reviews by [31,32,33]). Moreover, while differences in the aerobic and anaerobic glycolysis (CO_2_-respiration) may account for some pH differences between oxygenated and hypoxic regions of the tumors, differences in the proton and HCO_3_^−^ transport systems between the two regions have also been noted in an experimental model [34]. Our understanding of the precise mechanisms for the tumor’s lower extracellular pH (pHe) continues to be investigated and refined. The lower pHe, which is nearly universal, has been associated with higher rates of tumor cell division, enhanced invasiveness [35], increased metastatic potential of the tumor [36], alteration of the M1 to M2 macrophage phenotype [37], and increased cancer cell stem-like behavior [38]. Interestingly, neutralization of the low pHe of tumors with bicarbonate can reduce the metastatic potential of the tumor [39].

The pHe widely varies between non-necrotic tumors and is likely based on several interdependent factors, including the tumor cell proximity to the vasculature, the tumor size, the degree of hypoxia, and differences of glycolytic activity. For example, compared to normal tissues, the tumor pHe is relatively acidic, ranging from 6.4 to 7.2, with an average of about 6.8 [40,41]. Furthermore, there is heterogeneity of the pHe within a single tumor. The pHe within a breast tumor xenograft has been reported to vary between 6.4 to 6.8 [42]. While the pHe of tumors is more acidic than normal tissues, the intracellular pH of tumor cells is slightly more alkaline (pH 7.4 vs. 7.2). Thus, there is a reversal of the pH gradient in the tumor (i.e., extracellular to intracellular) compared to non-malignant tissues [33]. By alkalinizing the pHe of the tumor, investigators enhanced the efficacy of specific chemotherapeutic agents in tumor-bearing animal models [39,43].

In contrast, a low pHe of tumors may increase the efficacy of pH-sensitive nanoparticles. By increasing the positively charged amines on nanoparticles, the pH gradient established between the blood and normal tissues with a pH of 7.4 and the lower pHe of tumors may enhance the therapeutic specificity toward tumors [44,45]. Furthermore, increased protonation of weakly basic carriers/ligands in an acidic tumor matrix leads to greater binding and uptake by tumor cells, which have, in general, a higher negative surface membrane charge. An increase in uptake applies not only to basic carriers such as polyethylenimine (PEI), but also to nanoparticles modified with acidic peptides [18]. Additional interactions with pH-sensitive peptides/polymers may enhance the nanoparticle’s uptake. For instance, acidic peptides such as ATRAM adopt an α-helical conformation in mildly acidic environments, enabling the insertion and binding of the nanoparticle to the tumor cell membrane (and perhaps to other cells in the tumor) [27].

Once endosomal uptake occurs, disruption of pH-sensitive nanoparticles with release of the chemotherapeutic agent may occur in an acidic tumor endosome. In addition, the buffering properties of the pH-sensitive polymers may raise the pH of the acidic endosomes. Moreover, nanoparticles that disassemble and release a chemotherapeutic agent at the mildly acidic pHe of tumors have been developed. Whether this later mechanism occurs reliably in a tumor environment is debatable, but advances and fine-tuning of these carriers are being made.

In most solid tumors, administration of glucose (or using other pH lowering agents or methods such as hypoxia) usually reduces the pHe further by 0.2 to 0.4 pH units [40,46,47,48]. Some tumors, due to high glycolytic activity, had their tumor pHe reduced by as much 0.6 units [47,48]. Whether lowering pH with glucose can enhance the therapeutic efficacy of pH-dependent nanoparticles has not been investigated. While one study indicated that tumors did not become more aggressive by administering glucose [49], more studies are required to determine if intermittent reduction of the tumor pH enhances their malignant and metastatic potential. Alternative approaches, such as vasodilatory drugs (e.g., captopril), that lower tumor blood flow and reduce the pH could potentially be effective [50]. Still, temporal relationships between blood flow and tumor pH would be essential to determine the optimal accumulation for the nanoparticle. Moreover, regional embolization of blood vessels feeding the tumors (i.e., hepatomas) may lower tumor pH due to hypoxia and as a result, enhance the efficacy of a pH-sensitive delivery system [51].

The tumor pHe was measured initially by microelectrodes, but advances have been made with magnetic resonance imagining/spectroscopy (MRI/MRS) approaches. In contrast to microelectrodes which are both invasive and measure the pH at a single location [46,47,48], MRI/MRS is non-invasive and enables the heterogeneity of the tumor pHe to be measured [52]. In addition, these non-invasive techniques can potentially be improved to determine the pH of metastatic tumors in the lungs or liver. The resolution of MRI methods to assess spatial pH differences within tumors continues to improve and will likely guide future therapy of nanocarriers [52].

### 2.2. pH-Regulation within Tumor Endosomes

Although a lower tumor pHe may enhance the specificity and uptake of pH-sensitive nanoparticles, its mild acidity may not be sufficient to disrupt the nanoparticle and completely release the drug. Compared to the pHe of tumors, endosomes are markedly more acidic, with a pH range between 5 and 7.0. Because endosomes progressively become more acidic, they have a key role in disassembling pH-sensitive bonds and nanoparticles. Both these pH-dependent approaches can readily release a chemotherapeutic drug circumventing a significant problem of drug delivery systems [53]. Because leakage of small molecules from endosomes may occur [54], chemotherapy agents, once released from the pH-dependent nanoparticles, could potentially reach the cytosolic or nuclear target. The size and “leakiness” of endosomes are primary factors in determining lysis. In the presence of pH-buffering polymeric nanoparticles, HeLa cells with smaller endosomes and less leakiness were prone to lysis and release of relatively large molecules (i.e., oligodeoxynucleotides, plasmids) compared to ARPE-19 cells with larger and leakier endosomes. However, calcein, a small fluorescent molecule, readily leaked (without lysis) from endosomes of both cells transfected with the pH-dependent polyplexes. In addition to increasing endosomal leakiness, the pH-destabilizing nanoparticles have a second important function in lysing the endosomal membrane. Endosomal lysis by pH-buffering nanoparticles with release of small and large molecules occurs by osmotic lysis, by a detergent-like interaction with the membrane, or by both mechanisms [55]. The leakier the endosome, the less likely the osmotic lysis mechanism will occur. “Leakiness” vs. endosomal lysis by the detergent-like mechanism appears to be a matter of degree of the created pore size in the membrane. Although endosomal lysis may not always be critical for the escape of small drugs, it is likely necessary for the escape of siRNA or plasmids.

There has been a great deal of effort in developing pH-dependent nanoparticles and targeting specific endosomal pathways in tumor cells with ligand-conjugated nanoparticles. Specific endosomal markers or inhibitors are often used to determine the specific endosomal pathway of entry for the nanoparticle in vitro, but their use in vivo is limited. Nevertheless, competitive assays with an excess ligand can establish the importance of its cognate receptor in the cellular entry of the nanoparticle in vitro [56,57,58]. In vivo, targeted nanoparticles have frequently been demonstrated to have greater antitumor efficacy, presumably because of greater cellular uptake [59]. Upon entry, pH-disrupting nanoparticles disassemble in the progressively acidic endosomes or lysosomes. Still, there has been limited research understanding tumor endosomes, particularly with nanoparticles, beyond their becoming more acidic before merging with lysosomes [60]. Targeting the endosomal pathways of tumors in vivo has been primarily limited to conjugating ligands to nanoparticles [57,59,61,62]. Moreover, few studies have examined the differences between endosomes of tumors and normal cells (see review by Ko et al. [60]).

To target endosomes more effectively with pH-sensitive nanoparticles, it is important to comprehensively study tumor endosomes, their pathways, and qualitative and quantitative divergences with normal cells. Both normal and tumor cells contain the same endocytic pathways for entry of nanoparticles, and their endosomes perform molecular trafficking and sorting, which are regulated by the Rab family of small GTPases [60,63]. Once formed in these cells, most early endosomes ultimately merge with lysosomes resulting in the degradation of their exogenous contents such as nucleic acids. Alternatively, some endosomes may merge with multivesicular bodies, recycle to the surface with ligands, or target organelles such as the trans-Golgi network within cells. One area that requires investigation, particularly for pH-disrupting nanoparticles, is to determine differences in the pH of endosomes between cancer and normal cells.

Similar to those of normal cells, early endosomes of tumor cells become progressively more acidic. The V-ATPase proton pump is primarily responsible for acidifying endosomes, regardless of the endosomal pathway [60]. To compensate for the positive charge buildup within endosomes caused by the V-ATPase pump, chloride channels enable a passive influx of the ion. Notably, the chloride channels and sodium-hydrogen transporters (NHE) also have important roles in modifying the pH and charge potential of endosomes. These regulators closely coordinate with one another to fine-tune the endosomal pH. Whereas NHE 1–5 transporters, particularly NHE1, are usually associated with the cell membrane and acidification of the extracellular tumor environment, NHE 6–10 transporters are associated with endosomes. Parallel to the pHe of tumors, evidence is emerging that the pH of tumor endosomes drives a malignant phenotype. Interestingly, the pH of endosomes/lysosomes of tumor cells and the extracellular matrix closely interact to enhance the aggressiveness of the malignancy [26]. The tumor endosomal pH has also been linked to cancer cell proliferation, enhanced migration, reduced sensitivity to chemotherapy and radiation, and decreased patient survival. Epidemiological studies have established a strong association between one sodium-hydrogen transporter (NHE-9) and the poor prognosis of patients with esophageal cancer [64]. The gene for the alkalinizing NHE-9 transporter frequently is amplified in several cancers, including esophageal cancer.

Alteration in endosomal pH is complex and likely dependent on the type of malignancy and prior chemoradiation treatment. Transformation of fibroblasts by the ras oncogene has been associated with reduced acidity of endosomes [65]. Moreover, increased endosomal expression of NHE-9 in glioblastomas alkalinizes the endosomal lumen to about pH 6.5 [66]. This relative alkalinization reduced the EGFR receptor’s degradation with greater expression on the cell surface. Nevertheless, the picture that tumor endosomes are significantly less acidic than their normal counterparts appears far more nuanced and complex. For example, cancer cells treated under hypoxic conditions have endosomes that are “hyper-acidified” [67]. With more acidified endosomes, these hypoxic cells may entrap the weakly basic Dox (pKa, 8.3) released from nanoparticles more efficiently, preventing the drug from reaching its nuclear target [60,68]. Moreover, Fan et al. demonstrated that a rat glioma line has endosomes that overexpress the acidifying NHE-5 transporter. Although NHE-5 transporters are usually expressed on the plasma membrane, their location on early endosomes resulted in a lower pH and greater recycling of key growth receptors [69]. These somewhat conflicting studies could be partially resolved with further studies of patient-derived cancer cells to determine which of these mechanisms of endosomal pH is predominant. Regardless of the degree of acidity of endosomes, their lysis by pH-dependent nanoparticles, along with encapsulated drug release, may circumvent drug resistance.

Nonetheless, further delineation of mechanisms and pH differences in endosomal pH between malignant cells and normal cells may lead to more effective tumor-targeting carriers. We determined that pH-sensitive peptides carrying genes could target cells based on endosomal pH [70], and these findings may be extended to drug-loaded nanoparticles. When comparing normal and malignant cell lines transfection, branched pH-responsive peptides effectively transfected transformed or malignant cell lines but ineffectively transfected normal cells. While more investigations are required to determine if the endosomal pH of malignant and “normal” cells differ in tumors, targeting cancer cells (or other cells in the tumor) on their endosomal pH is an avenue that should be exploited. Based on these endosomal pH differences, intratumoral delivery of these pH-responsive NPs and strategies could be investigated. For systemic delivery, coupling ligand-directed therapies with intrinsic differences in the endosomal pathway of malignant cells could enhance the tumor specificity of the particle.

Significant technical hurdles still exist to measure the endosomal pH and lysis in vivo. In contrast, fluorescent imaging can readily measure the endosomal pH of cells in vitro. Numerous studies have determined the endosomal pH through the uptake of pH-sensitive fluorescent probes by endocytosis or preferential accumulation in acidic organelles. Nevertheless, we are unaware of systematically categorizing cells by their endosomal pH, including malignant cell lines. As oppose to such in vitro measurements, the pH of endosomes of tumor cells in vivo is very difficult to measure due to the inability of achieving sufficient amounts of a pH-sensitive probe in these organelles [52]. Until better methods are developed to measure the endosomal pH in vivo, there will be some uncertainty about the correlation between the in vitro and in vivo tumor models.

Like endosomal pH, several in vitro assays can evaluate endosomal lysis by pH-disruptive NPs [71,72,73,74]. The most common and easiest is a low molecular weight membrane impermeable fluorophore molecule, calcein (M.W.~677 kDa), added to the media that escapes into the cytosol upon endosomal lysis. The disadvantage of this method is that it is subjective, semi-quantitative, and may not distinguish leakiness from lysis. Recently, a reconstituted luciferase method identified the most effective carrier for lysing endosomes. This approach readily quantified the percent of endosomes that were lysed by the carrier [74]. Because the luciferase peptide fragment is small (M.W.~1940 kDa), most nanoparticles will incorporate the fragment without affecting the size or shape of the particle. Notably, less than 10% of the endosomes per cell released the hydrophilic peptide fragment.

Without further refinement, it is doubtful that these in vitro methods for determining endosomal leakage or lysis will have utility for tumors in vivo. Currently, endosomal leakage/lysis mediated by the nanoparticle is indirectly measured in vivo by determining the cytosolic/nuclear levels of the drug (or fluorophore, radioisotope) in the tumor or the functional effect of an encapsulated drug. Unfortunately, most nanoparticles remain in endosomes and do not escape the endolysosomal pathway [74,75]. The inability to lyse endosomes limits the efficacy of nanoparticles carrying drugs and, in particular, nucleic acids.

## 3. PH-Sensitive Bonds

Previous studies have noted that the tumor’s pH may influence the efficacy of the cancer chemotherapeutic agent. For example, by lowering the pH of the media of cancer cells, the antitumor efficacy of camptothecin and its analogs is improved by stabilizing the lactone ring [43]. Alternatively, raising the extracellular pH of tumors with sodium bicarbonate (added to the drinking water) enhanced tumor uptake and the antitumor efficacy of weakly basic agents such as Dox in murine tumor models [76]. Based on these chemotherapeutic experiments [43,76] and early studies with nucleic acids [77,78], pH-sensitive polymeric nanoparticles have been developed to deliver chemotherapeutic agents with and without nucleic acids.

Alternatively, pH-sensitive bonds that release Dox and PTX from a carrier or nanoparticle have directly targeted the acidic tumor matrix and endosomes. The well-characterized covalent chemical bonds sensitive to cleavage under mildly acidic conditions include imines, hydrazone, oximes, amides, methylene bridges, ketals/acetals, and coordination bonds with transitional metals (see reviews by Deirram et al. [79], Zhuo et al. [80] and Yan and Ding, [28]). The hydrazone and imine linkages are the most common pH-sensitive covalent bonds between a carrier and a drug. Less well-characterized, pH-sensitive non-covalent bonds between carrier and drug have been frequently cited in the field. While selected pH-sensitive covalent bonds that have minimal effect on the carrier (or nanoparticle) will be reviewed in this section, these bonds and other pH-sensitive bonds (acetal/ketal and amides), which have a significant role in nanoparticle disassembly, will be discussed in subsequent sections.

### 3.1. Covalent Bonds

#### 3.1.1. Hydrazone Bonds

The pH-dependent hydrazone bond has been used to link aldehyde/ketone-containing chemotherapy agents to carriers. As a result, Dox has commonly utilized the hydrazone bond. Dox has been linked via the hydrazone bond to an array of carriers such as polymers, proteins, polymeric micelles, ionic micelles, and paramagnetic particles [81,82,83,84,85]. Although lability of the hydrazone bond at physiologic pH has been problematic [84,85], studies indicate that a hydrophobic environment may stabilize the linkage [86].

**Figure 3:**
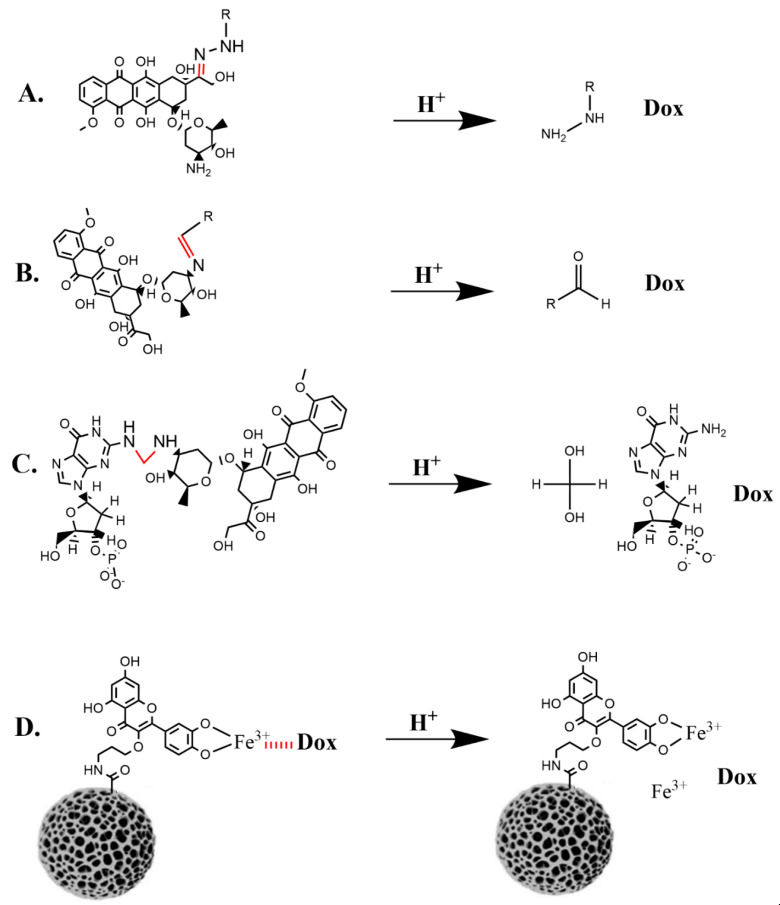
Selected pH-sensitive covalent bonds between Dox and carriers. (**A**) hydrazone [81,82], (**B**) imine [89], (**C**) methylene bridge between the exocyclic amino group of the guanine base and Dox [90], and (**D**) iron coordination bond. Upon exposure to mild acidity, release of Dox from the carrier occurs [91]. pH-sensitive bonds are highlighted in red and Dox in bold.

Lee et al. demonstrated that the hydrazone bond formed between Dox and the hydrophobic central core region of the polyester dendrimer was quite stable at pH 7.4 [81,82,83,84,85] (Figure 3). Notably, while 100% of Dox was released from the dendrimer at pH 5.0 in 48 h, only 10% was released at pH 7.4. Because the PEG-dendrimer-Dox carrier had a size of about 8 nm, preventing renal excretion, a half-life in excess of 24 h allowed more nanoparticle accumulation in tumors. In addition, the study also highlighted the advantages of pH-sensitive vs. insensitive bonds [81]. At the maximally tolerated dose of the hydrazone bond conjugate, the survival of the tumor-bearing mice was 100% at 60 days. In contrast, when Dox was attached to the dendrimer with a pH-insensitive carbamate bond, all mice died with a median survival of only 30 days. The reduced release of Dox in tumors from the pH-insensitive covalent bond than from the pH-sensitive bond probably accounted for the decreased antitumor efficacy. The inadequate release of the drug from pH-insensitive covalent bonds has plagued many studies.

More recently, a pH-sensitive (hydrazone) Dox lipid prodrug with high affinity to albumin had reduced toxicity, prolonged Dox circulation, and greater antitumor efficacy than Doxil^®^ [87,88]. Of the three lipids of the prodrug conjugate, the longest and most saturated stearic acid had the best result. Based on its apparent superiority to Doxil^®^, we think that approaches such as this will gain widespread clinical use prior to most other pH-strategies in this review.

Cross-linking polymeric micelles has been another method to stabilize micelles and increase their biological activity [82,83]. Nevertheless, cross-linking micelles may not prevent leakage of an encapsulated drug from the micelle before reaching its target. To improve retention of Dox in micelles, a hydrophobic derivative of Dox, methacrylamide Dox (Dox-Ma), was covalently linked via a pH-sensitive hydrazone bond. The cross-linked micelles comprised diblock copolymers (poly(ethylene glycol)-b-poly[N-(2-hydroxypropyl) methacrylamide-lactide) with the Dox-Ma derivative incorporated within the micelle core. Dox was released entirely within 24 h at pH 5 and 37 °C, whereas only about 5% was released at pH 7.4. The authors speculated that the hydrophobic environment, with its higher dielectric constant, likely slowed the release of Dox at the higher pH. Other investigators have also indicated that the rate of hydrazone bond cleavage depended on the groups surrounding the bond [86]. Moreover, the micelles with the Dox-Ma derivative showed improved anti-tumor activity and prolonged mouse survival than when treated with free DOX in mice bearing B16F10 melanomas [82,83]. Not only with this NP, we suspect that Dox and derivatives of Dox will require stabilization in most hydrophobic centers to deliver the drug effectively to tumors [92].

#### 3.1.2. Imine Bonds

A hydrophobic environment was also necessary to stabilize a pH-sensitive imine Schiff bond in a PEG-Dox conjugate [89]. Spherical micelles were studied, comprising curcumin and the PEG-Dox conjugate. The curcumin provided greater stabilization to the micelle through its hydrophobic interaction with the Dox component of the conjugate. In vitro assays demonstrated that approximately 90% of the Dox was released in 48 h at pH 5.0, whereas only about 10% was released at pH 7.4. Notably, in a mouse model with HepG2 tumors, a nanoparticle-containing Dox and curcumin had more apoptosis and antitumor effect than the combined free drug treatments or the nanoparticle containing only Dox.

Similarly, Dox and the OH radical-producing agent, aminoferrocene (Afc), were conjugated by a pH-sensitive imine bond to an oxidized derivative of hyaluronic acid (HA) [93]. The negatively charged HA has high affinity to the CD44 receptor, which is on the surface of many cancer cells. Moreover, HA-coupled therapeutic agents enhanced radical oxygen species and reduced the mitochondria membrane surface charge. About 60% of the Dox and aminoferrocene were released at pH 5 in 48 h, while only 15% of these agents were released at 7.4. In vitro experiments demonstrated that the HA- nanoparticle co-delivering these agents had a synergistic killing effect on 4T1 breast cancer cells.

#### 3.1.3. Methylene Bridges

Based on prior studies [94,95], pH-sensitive methylene bridge strengthened the Dox-DNA interactions. The methyl group of formaldehyde via an activated Schiff base intermediate forms a bridge between the amino group of Dox and the exocyclic amine of guanine. In the presence of excess calf thymus DNA, formaldehyde increased the half-life of Dox release from an aptamer to 8 ½ hours, whereas without formaldehyde, the half-life for Dox release was less than 5 min [95]. In a separate report, a rolling circle Dox-loaded nanoparticle stabilized by a methylene bridge released Dox readily in acidic environments [90]. Specifically, whereas 80% of the Dox was released at pH 5.4 in 3 ½ hours, only 20% was released at pH 7.4. Importantly, the methylene bridge likely did not affect the structural integrity of the nanoparticle or conjugate. In contrast, pH-sensitive acetal and ketal bonds, which are related to formaldehyde hydrates, have played a significant role in maintaining the structural integrity of nanoparticles and will be discussed in Section 5.

#### 3.1.4. Coordination Bonds

Coordination bonds indirectly [96] or directly [91] have a role in Dox release from the nanoparticle. Liu et al. developed a quercetin-modified mesoporous silicon nanoparticle to deliver Dox to breast cancer cells [91]. Dox was attached to surface-bound quercetin by a pH-sensitive iron coordination bond. About 80% of Dox was released from the nanoparticle in 24 h at pH 5, whereas only 15% of Dox was released at pH 7.4. Quercetin also enhanced the nanoparticle’s uptake and reversed multiple drug resistance. Consequently, the quercetin-Dox nanoparticle showed marked inhibitory activity in vitro toward both sensitive and resistant cells equivalent to the combined free Dox and quercetin treatment. With the coordination bond located on the exterior of the particle’s surface and exposed to competitive components of the blood (e.g., amino acids such as histidine and cysteine), this bond is unlikely to be sufficiently stable. In contrast, pH-sensitive coordination bonds located within the NP will probably be stable in the bloodstream [96].

### 3.2. Non-Covalent Interactions

A diverse number of carriers of chemotherapy agents have been reported that have pH-sensitive non-covalent interactions. These non-covalent bonds have been found between chemotherapy agents and carriers, including DNA, cyclodextrin, and carbon dots [97,98,99,100]. These bonds are primarily ionic but include supporting roles from hydrogen and hydrophobic bonds [101]. In addition, several investigators have credited increased protonation of Dox with higher solubility at acidic pHs for release from the nanoparticle. However, in some cases, the mechanism of the pH-related release remains unclear. It is improbable that these non-covalent interactions will be stable enough for clinical use without further stabilization, such as the DNA-Dox interaction with formaldehyde (Figure 3) [94,95].

## 4. Charge–Charge Repulsion with the Release of Hydrophobic Drugs

pH-sensitive polymers that make up nanoparticles are an attractive approach for delivering chemotherapy agents selectively to acidic environments such as the tumor matrix and endosomes [79]. These pH-responsive polymers contain weakly acidic or basic groups and have inspired advances in drug delivery systems. The polybasic polymers include poly(2-(dimethylamino)ethyl methacrylate (PDMAEMA), poly(2-(diethylamino)ethyl methacrylate) (PDEAEMA) [73,102], poly(2-(diisopropylamino)-ethyl methacrylate (PDPA or PDPAEMA) [103], poly(beta-amino ester) PBAE [104], polyethyleneimine (PEI), and poly-L-histidine (PLH) [105] or polyacidic polymers such as polyacrylic acid [106]. Although several pH-dependent polymers, such as PBAE, PLH, and polyacrylic acid, are biodegradable, only PBAE rapidly degrades upon exposure to mildly acidic pH. In contrast to pH-sensitive bonds that affect drug release in the tumor, pH-dependent polymers may enhance accumulation of the NP within the tumor.

**Figure 4:**
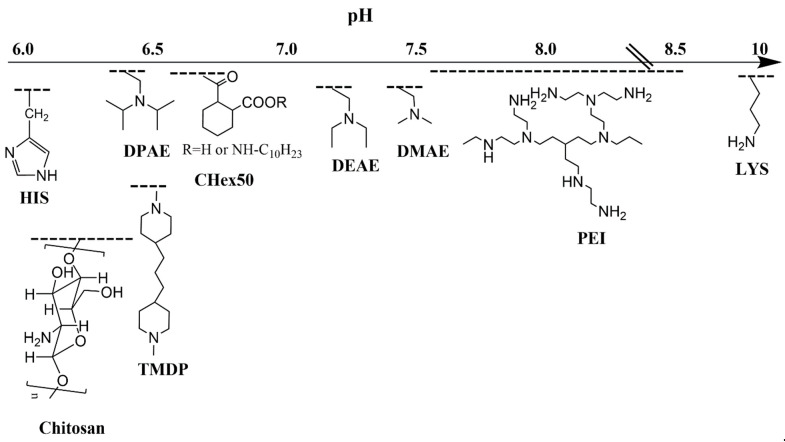
pKa of common pH-responsive groups in polymeric nanoparticles. Dotted line above the pH-sensitive group represents the approximate pKa of the functional groups. The length of the polymer and the presence of PEG can frequently alter the pKa of the pH-responsive groups [105,112].

The pH-buffering polymers (PEI, chitosan, polyhistidine, PDPAEMA, PDEAEMA, and PDMAEMA) have much in common, being weak buffering polybases and disrupting nanoparticles under acidic conditions. Nevertheless, PDMAEMA (pKa~7.4) and PEI (pKa~8.2 to 9.94 vs. multiple pKa with 55% protonated at pH 7.4) polymers carry a higher positive charge than PDPAEMA (pKa~6.4) and imidazole-containing polymers (pKa~6.0) at physiologic pH, which may affect their location in the NP [107,108,109,110,111] (Figure 4). More protonated polymers, such as PEI and PDMAEMA, are frequently located on the nanoparticle’s outer shell, whereas the largely unprotonated PDPAEMA and imidazole-containing polymers at physiological pH are in the hydrophobic core [109]. Nevertheless, the charge and hydrophobicity of a block co-polymer with a higher pKa may be altered, as discussed later (Section 4.2 and Section 5). Of note, the length of a pH-responsive polymer and the presence and length of a PEG block may significantly influence the pKa of the copolymer [105,112].

To take advantage of a low pH surrounding the tumor or in the tumor cell endosomes [30,63], the pKa of polymers making up micelles or nanoparticles are usually between pH 5 and 7.5. Thus, when the pKa of the polymeric molecule is near the pH of the surrounding milieu, the polymers become protonated within the particle, leading to charge–charge repulsion [113] with release of a hydrophobic chemotherapeutic agent. This disruption of nanoparticles within the acidic environment may occur extracellularly or intracellularly. Both these sites with different ranges of acidic pHs have been used to deliver hydrophobic drugs specifically to tumors. When siRNA or plasmids, together with chemotherapeutic agents, have been incorporated into nanoparticles, tumor endosomes are the primary target for release. Although charge–charge repulsion has a role in several parts of this review, the repulsion of charged polymeric nanoparticles has a primary role in drug release.

### 4.1. Release of Chemotherapeutic Agents

As the pH surrounding the nanoparticles is lowered, two biophysical boundaries are set for the particles. With repulsion of polymers in the particle, one boundary is a progressive increase in their size with decreased translucency, while the other boundary is complete disruption of the nanoparticles. The resulting outcome, an increase in size or complete disruption, depends on the extent of charge–charge repulsion, the attractive forces of the hydrophobic and hydrogen bonds between the particle polymers, and the polymer degradation rate. When hydrophobic domains such as PLLA (or PLA) are included in the copolymer, the particle will likely become progressively larger as the pH decreases to 5. Another factor determining whether the NP dissipates or enlarges, as the pH is lowered, is the polymer design (i.e., linear vs. graft). There are, of course, a range of changes that may occur between these two boundaries. For example, as the pH is gradually lowered between 7.4 and 5.0, the nanoparticle may increase in size before becoming smaller or disintegrating altogether [105]. Unlike polymeric micelles in subsequent sections, none of these pH-responsive nanoparticles in which the chemotherapeutic agent was solely incorporated were investigated for their antitumor efficacy in vivo.

The poly(β-amino ester) polymers (PBAE), formed by Michael’s addition reaction with different tertiary diamines, were one of the earliest developed pH-sensitive polymers (Table 1) [114]. A trimethylene dipiperidine-based PBAE with a pKa between 6.5 and 6.9 [104,115,116] has shown promise in delivering anticancer drugs such as PTX [117]. By blending Pluronic F-108 (PEG-PPO-PEG) and PBAE, stable PTX-loaded nanoparticles were formed by the solvent evaporation method [114]. These particles maintained their size of 110 nm and ZP of +30 mV with repeated freeze-drying and reconstitution. While the Pluronic F-108 component was not biodegradable, there was rapid degradation and dissolution of PBAE at pH 6.5. As the pH was lowered, the PBAE segment of the nanoparticle became progressively protonated and degraded, resulting in the complete destabilization of the nanoparticle and release of the drug. Consistent with reduced stability in an acidic endosomal compartment, the F-108/PBAE nanoparticles containing PTX showed increased cytotoxic activity compared to the non-pH-sensitive nanoparticles. All PBAE polymers in this review contained multiple subunits of the tertiary amine, trimethylene dipiperidine (TMDP).

While Pluronic F-108 was necessary initially to stabilize the nanoparticle polymer, Kim et al. formed a stable micelle nanoparticle with the diblock PEG-PBAE copolymer [115]. Using a similar strategy, Zhao et al. made a nanoparticle comprising the diblock polymer, D-α-tocopheryl-PEG(TPGS)-PBAE [116]. D-α-tocopheryl, a vitamin E derivative, has demonstrated potent inhibition of the MDR transporter, thereby reducing the efflux of hydrophobic chemotherapeutic agents. Docetaxel (DTX) was incorporated within the hydrophobic unprotonated PBAE segment of the particle. Consistent with biophysical properties of the PBAE polymer, the nanoparticle was a milky emulsion at pH 7.4, but was clear at a pH less than 6.4. The DTX-loaded nanoparticle showed increased cytotoxicity with both drug-sensitive and -resistant ovarian cancer cells compared to free-DTX. A marked enhancement of apoptosis by resistant cells was also observed with DTX-loaded particles compared to the free drug. Both increased intracellular accumulation of DTX and inhibition of the MDR transporter were the primary mechanisms of enhanced sensitivity of resistant cells to drug-loaded nanoparticles compared to free-drug. While the micelle showed a pH-dependence release of DTX, the micelle released nearly 50% of the drug at pH 7.4 within 48 h. This high release of Dox at physiological pH will probably limit the use of this micelle due to instability in vitro and in vivo.

**Figure 5:**
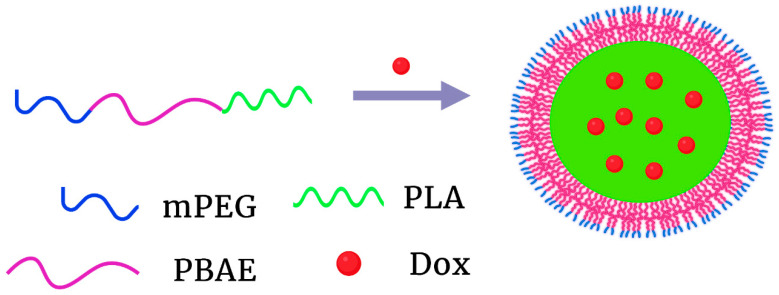
pH-dependent nanoparticles formed with the triblock polymer, mPEG-PBAE-PLA. While the block PBAE component was pH-responsive, the PLA hydrophobic core stabilized the particle and incorporated Dox [104].

More recently, a nanoparticle formed with the triblock polymer, mPEG-PBAE-polylactic acid (PLA), showed marked pH dependence [104] (Figure 5). With acid-base titration, the pKa of the copolymer was calculated to be 6.5. Unlike other PBAE-containing nanoparticles previously discussed that disintegrated, this triblock PBAE polymeric nanoparticle increased in size as the pH was lowered from pH 8 to 5. While the particle released only 20% of the Dox at pH 7.4, more than 80% of Dox was released at pH 6.0 during 48 h. More impressively, 96% of Dox was released at pH 5.0. Unfortunately, in vitro or in vivo cytotoxicity for this particle has not been evaluated for cancer cells. Not surprisingly, dynamics particle simulation at several pHs demonstrated that the PLA and PBAE formed an inner hydrophobic core and were essential to incorporate the unprotonated Dox. Because of the complexity of many copolymer structures, this relatively simple triblock polymer is promising. However, Dox would likely leak from this particle into the vessels without further stabilization of the core.

Several block and graft polymers containing PDEAEMA have been synthesized to form nanoparticles [102,118,131]. The pKa of the PDEAEMA block, which is influenced by the length of the PDEAEMA and PEG block, has ranged between pH 6.9 and 7.44 [112]. Chen et al. prepared mixed micelles with a pH-sensitive poly(2-(diethylamino) ethyl methacrylate) (PDEAEMA)-based copolymer [118]. Different combinations of mixed diblock and triblock copolymers (mPEG—b-PDEAEMA-b-(poly-(methylmethacrylate) (PMMA); PDEAEMA-b-PMMA; and (poly(poly(ethylene glycol) methyl ether methacry-late (PPEGMA)-b-PDEAEMA) exhibited a low critical micelle concentration ranging from 1.95–5.25 mg/L. The optimal micelle had a size of less than 100 nm, an entrapment efficiency of 55%, and a loading capacity of 24%. Whereas the PEG, PPEGMA, and PDEAEMA domains provided a hydrophilic shield for the micelle, the PMMA (poly-(methyl methacrylate)) polymeric domain was hydrophobic, enabling the incorporation of Dox. Compared to the single block copolymeric micelle, the optimal mixed micelle comprising mPEG-bPDEAEMA-PMMA and PDEAEMA-b-PMMA copolymers showed improved pH-responsiveness. These mixed micelles released about 20%, 50% and 80% of Dox at 7.4, 6.0 and 5.0, respectively, within 60 h. Probably due to incomplete release at 48 h, the mixed Dox-loaded micelles were less cytotoxic to HepG2 tumor cells than free Dox.

Instead of a linear block copolymer, Feng et al. synthesized a 3-armed block polymer that emanated from a central cholate (CA) core [119]. The block polymer comprised poly(ε-caprolactone)(PCL)-b-PDEAEMA-b-PPEGMA. The hydrophobic PCL domain incorporated PTX, while the PPEGMA formed a hydrophilic shield around the micelle. In addition to the micelles showing good encapsulation efficiency of 48.2% and drug loading capacity of 29.9%, they demonstrated pH-dependent behavior under acidic conditions. Their size increased modestly from 268 nm to about 290 nm as the pH was lowered from 7.4 to 5.0. Furthermore, the release of PTX was 20% and 55% at pH 7.4 and 5.0, respectively, in 80 h. For intermediate dosages of PTX, there was a trend suggesting that pH-dependent PTX-loaded micelles had greater cytotoxicity toward mouse-transformed NIH-3T3 cells than non-pH-dependent micelles after 48 h [119]. With the modest increases in the sizes as the pH was lowered, the micelles perhaps were too stable to exhibit distinct differences between pH-dependent and -independent micelles. In marked contrast, some nanoparticles comprising= multiarmed copolymers demonstrated dramatic pH differences in size and drug release, as later discussed [123].

A closely related polymer to PDEAEMA but with a lower pKa of 6.4 is PDPAEMA [110]. Liang et al. incorporated the PEG-Dox conjugate with the H4R4 peptide in a hydrophobic PDPAEMA micelle [103]. While the PDPAEMA polymer was responsible for releasing the PEG-Dox conjugate at acidic pH, the H4R4 peptide significantly enhanced the disruption of the endosomal membrane and cytosolic delivery of the conjugate. The incorporated H4R4 co-peptide increased the cytotoxicity of Dox-loaded nanoparticles by 30-fold compared to those without the co-peptide. In contrast to most nanoparticles, these Dox-loaded nanoparticles were nearly 16-fold more cytotoxic to drug-sensitive HeLa cells than free Dox (IC_50_, 0.063 vs. 1 μM). Moreover, these Dox-loaded nanoparticles were stable and released about 10% of the Peg-Dox at pH 7.4, while the nanoparticles released about 90% of the PEG-Dox at pH 5.5 during the same time (thirty-six hours). It is unclear where the H4R4 peptide is located since it could be disruptive to the nanoparticle, but we assume that the R4 segment of the co-peptide is on the surface. Consequently, we doubt this particle would be stable in vivo without a longer hydrophobic histidine segment (i.e., R4H10).

Compared to PDPAEMA micelles, micelles formed with a diblock DEAEMA:DPAMA copolymer enabled more escape of small and large molecules in the acidic tumor matrix, probably because of the higher pKa. By incorporating different amounts of DEAEMA and DPAEMA randomly (r) into a copolymer (P(DEAEMA-r-DPAEMA), Kongkatigumjorn et al. made micelles that would disassemble at different pHs [120]. The micelles’ inner core comprised PDEAEMA, P(DEAEMA-r-DPAEMA (3:1), P(DEAEMA-r-DPAEMA (1:1), P(DEAEMA-r-DPAEMA) (1:3), or PDPAEMA completely disassembled at pH 7, 6.6, 6.2, 5.8, and 4.9, respectively. Dissolution of the micelles was consistent with the pKa for the PDEAEMA, PDPAEMA, and the random copolymers. Except for the size of PDPAEMA-only micelles, which increased significantly before disassembly, the size of the other micelles modestly increased before disassembly. The antitumor efficacy of these micelles loaded with Dox would be interesting to examine.

With a pKa of 7.5, it is not surprising that the PDMAEMA polymer block is on the exterior surface of micelles. Car et al. [121] developed pH-responsive micelles based on diblock copolymers comprising the hydrophobic polydimethysiloxane (PDMS) with a fixed length (74 monomeric units) and PDMAEMA of various lengths. They determined that empty micelles of PDMS and PDMAEMA with five monomeric units (AB5) had significantly lower toxicity compared to PDMAEMA copolymers with greater lengths (13 or 22). Moreover, Dox-loaded AB5 micelles, when exposed to pH 5.5, released about 90% after 2 days, while they only released about 15% at pH 7.4. As expected, the size of the micelles and zeta potential increased as the pH was lowered. In general, as in this case, we think it is preferable for the partially protonated component with higher pKa, such as PDMAEMA, to be on the external surface of the pH-responsive NP. The PDMAEMA component within the hydrophobic core may be problematic with unwanted drug leakage in animal models.

In addition to PBAE polymers, histidine-rich polymers were among the earliest used to form nanoparticles [105,122,123,132,133,134,135,136,137,138,139,140,141,142,143,144,145,146,147,148]. A primary reason for their development was that histidine(imidazole)-rich polymers were effective and relatively non-toxic in forming polyplexes for gene therapy [149,150]. Consequently, a large number of diverse structures of these polymers have been synthesized to form nanoparticles. Micelles, mixed micelles, and nanovesicles have been made from block linear and graft polymers. Since this topic has been reviewed recently, we will discuss only a few notable studies utilizing these polymers (see review by Imitaz et al. [151])

A remarkably stable nanoparticle at physiologic pH, yet one that demonstrated marked pH-responsiveness, comprised a targeted PEGylated polyhistidine graft co-polymer (poly(itaconic acid)-g-FA-PEG-g-PLH) [122]. Less than 5% of Dox was released by 48 h at pH 7.5 and 37 °C, whereas about 90% was released by 48 h at pH 5. By lowering the pH, there was charge reversal (negative to positive) and a step-wise increase in the size of the micelles. Notably, these folate-targeted Dox-loaded micelles showed a greater reduction in cell viability over free Dox or non-targeted micelles. The complexity of this micelle makes it unlikely to reach clinical trials. Still, with its in vitro stability and pH-responsiveness, this particle’s in vivo pharmacokinetics and antitumor efficacy should be fully investigated.

Like the 3-armed polymer containing PDEAMA [119], a 5-armed PLGA copolymer was synthesized containing a single histidine at the end of each arm [123]. In contrast to the 3-armed nanoparticles, the 5-armed histidine nanoparticles showed marked increases in size in low pH media. The histidine-containing particles increased in size from 157 nm at pH 7.4 to 1268 nm at pH 6.5. Consistent with an increase in size, the release of both drugs (Dox, disulfiram) was pH-dependent. Despite the increase in size of the nanoparticles at low pH, the particles showed greater penetration in a tumor spheroid model than control nanoparticles.

### 4.2. Dual Delivery of Chemotherapy and Nucleic Acids

The pH-buffering polymeric nanoparticles that co-delivered drugs and nucleic acids have many similarities to those that delivered only drugs. These include pH-dependent charge–charge repulsion accompanied by size changes and the release of drugs. Nonetheless, these dual therapeutic nanoparticles are more complex with redox-dependent linkages often incorporated. As a result, multilayer nanoparticles or micelles are required to effectively deliver and release dual therapeutic agents with differing biophysical properties to tumors.

Although more protonated polymers at physiologic pH, such as PEI, DMAEMA, and polylysine, are usually on the nanoparticle’s outer shell, these polymers become more hydrophobic upon binding to nucleic acids. Consequently, depending on the polymer and the polymer design, the type of nucleic acids, and the ratio of polymers to nucleic acids, pH-dependent polymers, notwithstanding their pKa, may be located in the inner, middle, or outer layers of the nanoparticles.

Regardless of where the pH-dependent polymers are located, these polymers have an essential role in disrupting nanoparticles in the tumor. For dual therapy delivery nanoparticles synthesized from blocked copolymers, tumor-inhibitory plasmids were confined to exterior surfaces, whereas the smaller siRNA may be in the inner or outer shells. Notably, the buffering of pH-dependent polymers is attenuated upon binding to nucleic acids [54]. The combination of siRNA targeting oncogenes and chemotherapy in the nanoparticle had a markedly greater antitumor effect in every study compared to delivering a single agent.

One potential advantage of pH-responsive polymers on the nanoparticle’s exterior is that the particle’s increasing positive surface charge in acidic environments enhances binding and uptake by cancer cells. There have been several examples of pH-dependent copolymers that are part of the outer shell of the nanoparticles. Davoodi et al. formed a dual pH- and redox-responsive micelle with a triblock copolymer of PEI-poly(ε-caprolactone)-PEI (PEI-PCL-PEI) to deliver the p53-expressing plasmid and Dox [124]. In addition to binding to the p53 plasmid, the partially protonated PEI component on the outer shell of the micelles likely has another vital role. In a low pH environment, greater protonation of PEI would be expected to disrupt the nanoparticles, facilitating the release of the plasmid and Dox. Nonetheless, the pH-responsiveness of Dox release from the particle was not measured, but reducing conditions (DTT, 10 mM) showed a modest enhancement of Dox release. Like other hydrophobic drugs, Dox was incorporated in the hydrophobic domain (i.e., poly-caprolactone) of the micelle (Table 1). Notably, PEI is not metabolizable and can be toxic depending on the degree of branching and molecular weight. Although the low molecular weight branched PEI that formed NPs (without drug or p53-plasmid) showed minimal cytotoxicity, PEI should be avoided in most cases to formulate NPs carrying chemotherapeutic agents. Nevertheless, PEI provides a paradigm for developing copolymers with similar but less toxic properties.

**Figure 6:**
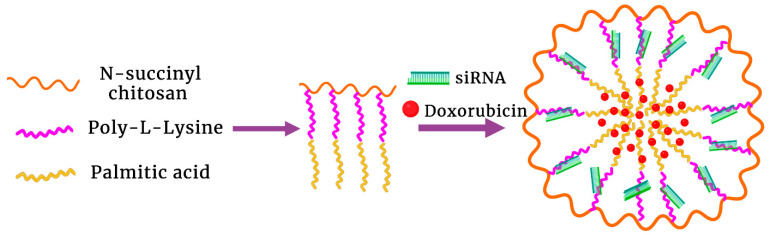
The pH- and redox-sensitive micelles formed with graft triblock succinyl chitosan-polylysine-palmitic acid copolymers. While the chitosan outer layer was pH-responsive, the polylysine middle layer was neutralized by siRNA, and the palmitic acid inner core incorporated Dox [125].

In another study, Zhang et al. synthesized a graft triblock succinyl chitosan-polylysine-palmitic acid copolymer to form micelles, incorporating Dox and siRNA targeting the Pgp transporter (siPgp) [125] (Figure 6). Chitosan, a pH-dependent polysaccharide with a pKa between 6.0 and 6.5 [152], was on the particle’s outer shell, and the nucleic acid was primarily bound to the highly charged polylysine (pKa~10.3) in the middle layer. Consequently, the increase in the ZP and size of micelles at lower pHs likely resulted in their instability and release of Dox from the inner hydrophobic palmitic acid layer. The siPgp and Dox-loaded micelles inhibited HepG2 tumors in vitro and in vivo more than free Dox alone. The efficacy of this dual therapeutic micelle compared to the other therapies was particularly impressive in vivo in a mouse model with HepG2 tumors that expressed high levels of the Pgp transporter.

Although these first two studies showed evidence suggestive of the pH dependence release of the drug (i.e., increase in size and ZP), the authors did not measure the drug and nucleic acid release from the nanoparticle at lower pH. Consequently, the question still arises whether pH-dependent polymers located on the outer shell are more effective at disrupting the particle and releasing the drug and shRNA compared to polymers located inside the particle. This cannot be definitively answered, although the pH-dependent polymer on the outer shell of the particle in the following study demonstrated an impressive release of the drug and shRNA [126]. Moreover, validation that a pH-dependent polymer on the outer shell has an important role in disrupting the nanoparticle and facilitating the release of Dox was also shown with PDMAEMA-containing micelles [121].

A third study with a pH-responsive polymer component on the outer shell exhibited a dramatic release of shRNA and drugs as the pH was lowered [126,127]. The nanoparticle carrier of PTX and an shRNA plasmid targeting Twist (shTw) comprised a poloxamer-PEI conjugate and a stabilizer polymer, D-alpha-tocopheryl polyethylene glycol 1000 succinate (TPGS). While the PEI component was on the exterior shell and interacted with the shRNA plasmid, the hydrophobic tocopherol component stabilized PTX within the inner core of the particle. The shTw and PTX exhibited pH-dependence release from the nanoparticle at pH 6.5 and 5.0. Indeed, greater than 80% of both were released at pH 5.0 in 72 h at 37 °C. A nanoparticle carrying shTw and PTX markedly reduced the pro-metastatic Twist factor and reduced the viability of 4T1 cells compared to controls. Building on these results, the therapeutic nanoparticle demonstrated increased accumulation of the shRNA and PTX in tumors in vivo more than free-PTX. Moreover, nanoparticles that co-delivered shTw and PTX eliminated lung metastases and synergistically reduced the growth of the primary 4T1 tumors by 80% compared to the free PTX-treated control.

There have been several nanoparticle designs in which the pH-responsive polymers were not on the exterior of the nanoparticle. Similar to a previous discussed study [125], siRNA was in the middle layer. Still, the pH-sensitive polymer here was part of the inner instead of the outer layer of the particle [128]. Chen et al. designed a dual responsive carrier with pH- and redox-sensitivity to deliver Dox and siRNA. Micelles were formed with a triblock polymer PEG-b poly(N-(2,2′-dithiobis(ethylamine)) aspartamide) (PAsp(AED))-b-PDPAEMA. Whereas the inner core consisted of the pH-dependent PDPAEMA and the outer core comprised PEG, the middle layer consisted of a redox-dependent PAsp(AED) domain. The inner shell retained the Dox, whereas the middle shell contained the BCL-2 siRNA (siBCL-2), reversing Dox resistance and promoting tumor cell apoptosis. With a pKa of 6.4, the pH-dependent yet hydrophobic PDPAEMA co-polymer is ideal for the inner core. One interesting aspect was that the release of Dox from the micelle was minimal (~5%) at 7. 4 and 37° over 24 h. The siRNA in complex with the middle shell formed a compact layer, and upon exposure to glutathione and a pH of 5, Dox and siRNA were rapidly released. Concomitant with release, the micelles increased significantly in size from about 64 nm at pH 7.4 to 455 nm at pH 5.0. In addition, the micelles carrying Dox and siBCL-2 showed enhanced tumor accumulation with marked synergistic antitumor activity in vivo. Because the unwanted release of Dox at physiologic pH may be problematic for many micelles, incorporating a middle layer with a siRNA may mitigate these instability issues [128,153]. Barring synthetic scale-up problems, this micelle with a pH-sensitive yet hydrophobic center represents one of the most promising candidates.

Gao et al. synthesized PEG-b-PLA-polyhistidine-ss-oligoethylenimine (PEG-b-PLA-PLH-ss-OEI) polymer, forming a redox- and pH-sensitive nanoparticle to deliver Dox and MDR-targeting siRNA [129]. PEG formed a hydrophilic shell, whereas the PLA and PLH domains formed the Dox-incorporated hydrophobic core. In contrast to other micelles, the siRNA was bound to a positively-charged OEI component (a subunit of PEI) in the inner cavity. Regarding pH-dependency, the nanoparticles released about 40% of Dox at 7.4 in 12 h, while they released about 90% of Dox at pH 5.5. In contrast to Dox release, siRNA was both pH- and redox-dependent. Notably, in vivo studies showed that these dual-delivery nanoparticles completely inhibited the growth of drug-resistant MCF7 xenografts. Unlike larger molecular weights of PEI, OEI would be expected to be significantly less toxic. Nevertheless, scale-up of these NPs, in which the partially protonated OEI component is internally located and neutralized with nucleic acids, may be challenging.

In addition to solid tumors, other diseases such as rheumatoid arthritis and osteoarthritis may also have a low pH (i.e., in the synovial fluids), so pH-sensitive nanoparticles could potentially target these pathological conditions [154,155]. Zhao and Zhang examined whether a PEG-PEI/PEI-PCL nanoparticles incorporating methotrexate and Notch-1 siRNA would decrease inflammation and paw thickness in a rheumatoid arthritis model [130]. The size and ZP of these nanoparticles were 162 nm and +22.4 mV, respectively. Despite the positive ZP, the half-life of the drug-loaded nanoparticle in the blood stream was six hours. Concomitant with improved pharmacokinetics, the PEI nanoparticle, delivered systemically, reduced paw thickness markedly more than methotrexate alone. Although the authors did not examine the release kinetics of methotrexate or the siRNA at low vs. physiological pH with these carriers, the charge–charge repulsion and osmotic swelling of PEI polyplexes have been well-established for the release of drugs into the cytosol. Negatively charged methotrexate was electrostatically attached to the exterior surface of the positively charged PEI polyplexes, whereas the siRNA neutralized PEI to varying degrees throughout the particle. Interestingly, the methotrexate PEI polyplexes did not aggregate red blood cells, whereas the PEI polyplexes did [156].

## 5. Disassembly of Nanoparticles Couple to pH-Sensitive Covalent Linkages

We have discussed nanoparticles disassembly based primarily on charge–charge repulsion at lower pHs with concurrent release of chemotherapeutics [105]. Moreover, pH-sensitive linkages between chemotherapy and carrier, which had minimal effect on the structural integrity of the carrier, have also been discussed. We will now examine two approaches based on the combination of pH bond cleavage and the disruption of the nanoparticle to release the chemotherapeutic drug.

The first approach relies on the cleavage of pH-sensitive linkages affecting the integrity of the nanoparticle. This type of acid hydrolysis can result in a hydrophobic to hydrophilic phase change or dissolution of the polymeric structure, releasing the encapsulated drug. Second, acid-catalyzed cleavage of the bond between a hydrophobic molecule (including the drug) and the polymer may destabilize the nanoparticle, enhancing drug release. Concomitant with the release of the hydrophobic drug, the repellant charges on the polymeric nanoparticles become progressively dominant. Similar to chemotherapy and nucleic acid co-delivery systems, titrating hydrophobic molecules to counter the positive internal charge of copolymers, in many instances, may limit their clinical use currently [157,158]. The pH-sensitive bonds in these two scenarios have included acetals, imines, hydrazones, and zinc-imidazole coordination bonds.

**Figure 7:**
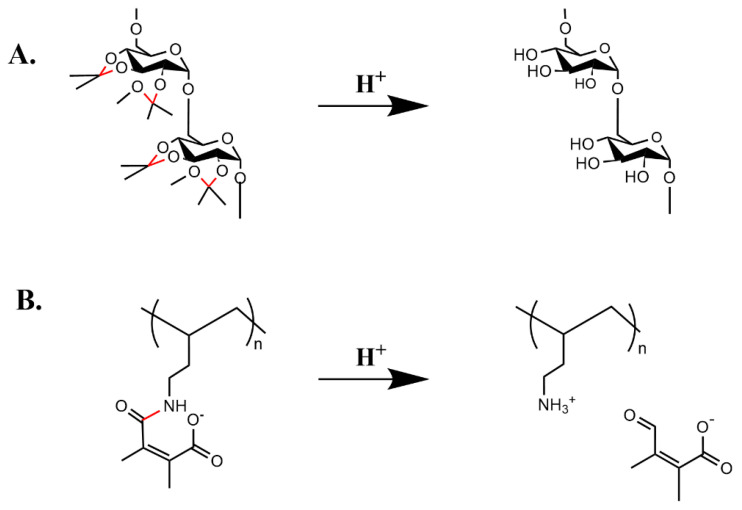
Disruption of nanoparticles based on pH-sensitive bonds. (**A**) Upon exposure to acid, the pH-sensitive acetal bonds broke apart, and the hydrophobic dextran nanoparticles underwent a phase transition to hydrophilic polymeric chains with release of acetone and methanol [159]. (**B**) With pH-dependent release of dimethyl maleic acid from the coating Peg-b-poly allylamine copolymer, the copolymer became positive with charge–charge repulsion from the positively surface-charge carbon dot. Consequently, cis-platinum on the carbon surface was then susceptible for release [165]. pH-sensitive bonds are highlighted in red.

In an early study, Bachelder et al. masked the hydroxyl groups of dextran with acetals, resulting in dextran particles becoming water-insoluble (Figure 7A) [159]. With a solvent evaporation method, the particles of acetal-derivatized dextran were formed with a size of about 240 nm. When exposed to a mildly acidic pH of 5.0, the acetal groups were removed with complete dissolution of the particles within 72 h. Furthermore, a fluorescent hydrophilic compound incorporated within the nanoparticles had a release half-life of 10 h at 37 °C and pH 5 compared to about 15 days at pH 7.4. This study led to other applications of pH bond cleavage affecting the polymeric carrier (Table 2).

Similarly, pH-sensitive acetal bonds affected the structural integrity of a PLLA delivery particle for chemotherapy [160]. In contrast to acetal-derivatized dextran nanoparticles, PLLA particles containing Dox were much larger, ranging in size from 2 to 15 μm. The 14 kD polymers that formed the nanoparticle consisted of multiple PLLA domains in which acetal bonds were inserted into the backbone. In these nanoparticles, Dox was released in a pH-dependent manner, and the particles showed significant toxicity toward breast cancer cells in vitro. Nevertheless, based on their large size and poor cellular uptake, the nanoparticles likely dissipated outside the cells with the release of the Dox. Compared to Dox-loaded nanoparticles that did not have a pH-dependent linkage, the acetal-enriched Dox nanoparticle injected intratumorally inhibited tumors significantly more in a mouse model. To establish their clinical potential, these nanoparticles should be compared with other drug-containing biomaterials, which have shown impressive antitumor efficacy when injected intratumorally [161].

By altering the balance between the hydrophobicity and the charge of the inner core of the micelle with a pH-sensitive bond, several studies have shown promise in delivering chemotherapeutic drugs. A significant problem has been the lack of retention of the hydrophobic drug within the pH-sensitive nanoparticle or micelle. In particular, micelles formed with the more protonated polybasic polymers such as DMAEMA (pKa-7.5) and polylysine (pKa~10.3) or the negatively charged polyacidic polymers (polyacrylic acid, pKa~4.5) in the hydrophobic domain may be destabilizing at physiological pH. As previously discussed, one solution to stabilize the positively charged polymers in the interior has been their interaction with negative-charged molecules such as siRNA.

Besides the addition of siRNA, increasing the hydrophobicity in the inner core may stabilize the micelle to deliver PTX or Dox. For instance, Qiu et al. synthesized a pH and redox-dependent star copolymer to form a micelle [158]. The branches consisted of copolymers of methacryloxyethoxy)-benzaldehyde) (MAEBA) and DMAEMA emanating from the 2,2′-dithiodiethoxyl dimethacrylate branch point. To incorporate the drug more stably within the micelle, Dox was covalently linked by an aromatic imine linkage to the aldehyde-containing MAEBA component of the polymer. Although imine bonds have been reported to be unstable at neutral pH, studies have demonstrated that the Pi–Pi hydrophobic interactions may stabilize the imine bonds. Moreover, despite the partially charged DMAEMA in the interior, the hydrophobic Dox and the benzyl groups enhanced the nanoparticle’s stability by increasing the interior’s hydrophobicity. After 48 h and at pH 7.4, the aromatic imine linkage with Dox was stable, with less than 5% of the Dox released, whereas about 50% of the drug was released at pH 5.0 and about 65% at pH 5.0 with 10 mM DTT, respectively. Notably, despite exposure to low pH and DTT, the incomplete release of Dox may limit its antitumor efficacy.

**Figure 8:**
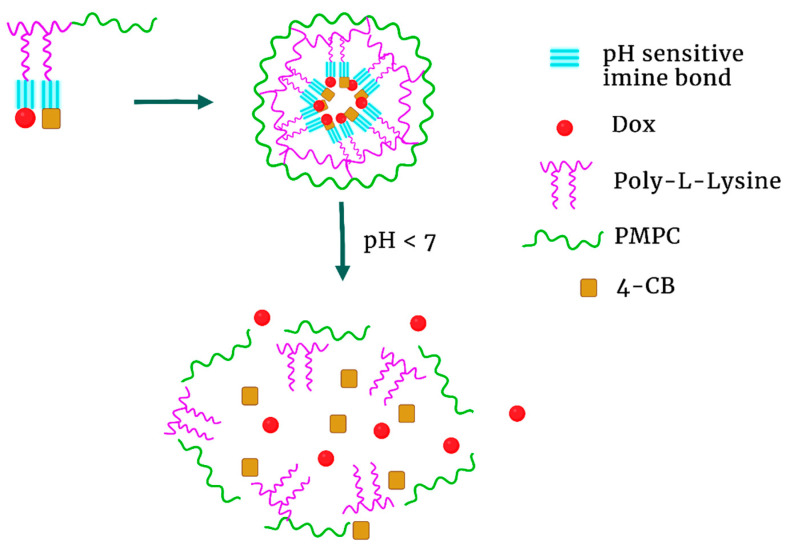
PMBC-b-polylysine copolymer-comprised micelles. The positive charge of polylysine was neutralized by conjugating a pH-sensitive imine bond with the hydrophobic molecules, 4-CB and Dox. With acidification, the micelles progressively enlarged, and 4-CB and Dox were released by pH-sensitive imine bond [157].

This strategy of increasing the hydrophobicity of the inner core was further investigated by modifying the highly charged poly-L-lysine (pKa~10.3). Ma et al. prepared polymeric micelles by conjugating 4-carboxybenzaldehyde (4-CB) and Dox with an amphiphilic copolymer via a pH-sensitive imine bond [157] (Figure 8). Dox and 4-CB attached to the polylysine block segment formed the hydrophobic core, whereas the zwitterionic poly(2-methacryloyloxy-ethyl phosphorylcholine) (PMBC) block formed the outer shell. When exposed to an acidic environment, the imine linkages were readily broken with the release of Dox and 4-CB from the polylysine segment. Release of Dox from the micelles at pH 7.4, 6.8, and 5.5 was 30%, 40%, and 70%, respectively (48 h). As these two hydrophobic molecules were released in acidic media, the micelle underwent a charge conversion (by unmasking the poly-L-lysine), size enlargement, and disruption. Over 24 h, the micelles’ surface charge changed from −12 at pH 7.4 to + 34 mV at pH 5.5. Compared to free Dox, the pH-sensitive micelle showed greater inhibition of initially large 4T1 tumors (~100 mm^3^) (*p* < 0.05) in mice and less organ toxicity. Prior to charge reversal, the negatively charged micelles may be less toxic and have a longer half-life, enabling greater amounts of the drug to reach the tumor. Still, because of the complexity in establishing a hydrophobic core by neutralizing the charge on the side chains of lysines by conjugation with Dox and 4CB, this NP would be particularly challenging to scale up to treat humans.

Using a different approach, Deng et al. also prepared micelles that reversed from a negative charge at pH 7.4 to a positive charge at pH 6.0 [162]. The pH-sensitive micelles comprised PEG-b-(PCL-co-γ-dimethyl maleamidic acid-ε-caprolactone) (PEG-b-(PCL-co-DCL) polymer. Under acidic conditions, these micelles underwent rapid charge reversal by hydrolysis of the acid labile-β-carboxylic amides (i.e., dimethyl maleamide). Within 2 h, the ZP of −7.0 of these micelles at pH 7.4 changed to +3.5 at pH 6.4. The negative charge on the β-carboxylic amide on the polyester block enhanced the loading capacity of the positively charged Dox in the micelle. Whereas 10% of the Dox was released from micelles at 7.4 in 12 h at 37 °C, nearly 90% was released at pH 5.3. The pH-sensitive PEG-b-P(CL-co-DCL) loaded micelles inhibited the cell viability of HepG2 cells more effectively than the non-pH sensitive micelles. The charge reversals of these β-carboxylic amide polyesters were similar to others with β-carboxylic amides such as citraconic-amide functionalized polymers [166]. The charge reversal, the stability, and the pH-responsiveness of this micelle are particularly attractive properties, but unfortunately, in vivo efficacy studies were not carried out.

Although the disassembly of nanoparticles has been primarily centered on the basic polymer repulsion, disruption of particles may also occur with charge–charge repulsion of polyacidic polymers. Moreover, in contrast to liposomal Doxil^®^, delivery systems for PTX have often been plagued by low loading and poor release of the drug. To overcome these potential problems, Gu et al. conjugated PTX to the block copolymer of PEG and poly(acrylic) acid (PAA) with an acid-labile acetal bond [163]. The loading capacity of these micelles for PTX was as high as 43%. While PTX-loaded micelles were quite stable at 4 °C for several months, the micelles released at 37 °C (48 h) about 29%, 66%, and 87% at pH 7.4, 6.0, and 5.0, respectively. With the release of hydrophobic PTX in the acidic environment, the repulsion of the negatively charged PAA chains became the dominant factor in increasing the size and destabilizing the micelle. For in vitro inhibition studies, the PTX-loaded micelle significantly reduced cell viability in drug-resistant A549 cells more than free PTX (IC_50_, 10.9 vs. 175.8 μg/mL). The simplicity of these diblock copolymeric micelles makes these appealing, but again as in the prior study, in vivo studies were not done. This study also highlights that drug release at pH 7.4 under simulated in vivo conditions does not necessarily correlate with long-term in vitro stability. Nevertheless, the higher the release of the drug at 37 °C, the more likely there will be reduced antitumor efficacy and more side effects.

Not all pH-dependent nanoparticles, which released hydrophobic drugs, were made up of copolymers. Liu et al. modified the sulfhydryl-rich keratin protein with iodoacetate and then hydrazine to form a pH-sensitive hydrazone linkage with Dox [164]. The keratin-Dox nanoparticle had a size of about 250 nm after desolvation and cross-linking with genepin. When exposed to a pH of 5, the keratin nanoparticle slowly released about 40 and 60% of Dox at 24 and 48 h, respectively. Only 5% of Dox was released from the keratin at pH 7.4 over 11 days. Moreover, there was a charge reversal of the keratin-particle. The ZP of the keratin-Dox nanoparticle at pH 7.4 and 5.0 changed from −30.4 to +6.7, respectively. Although charge reversion did not occur at an extracellular pH of 6.8, the reduced negative surface charge should enable the nanoparticle to be taken up more quickly by the cancer cell. Notably, these Dox-containing nanoparticles effectively inhibited human alveolar adenocarcinoma cells in vitro (A 549) and mouse hepatomas in vivo (H22 tumors). In addition, animal survival was prolonged in the H22 tumor-bearing mice treated with the nanoparticle compared to free Dox. [164]. The keratin-Dox nanoparticles also showed less toxicity, as evidenced by body weight during treatment than free Dox.

## 6. PH-Sensitive Coatings

Although there are mechanistic overlaps with previous sections, the pH-dependent coating distinctly differs from the remainder of the nanoparticle. In contrast to the pH disassembly of nanoparticles previously discussed, a lower pH does not usually increase the size of these cloaked NPs. In addition, coating nanoparticles with pH-sensitive polymers has three objectives. First, coating of the particle may increase the stability and reduce drug release at physiologic pH. Second, coating with a pH-sensitive polymer/peptide may enhance the targetability of the nanoparticle. Several of the coatings may increase the half-life of the NPs in the bloodstream.

Additionally, with the lower pHe in the tumor, the pH-responsive coating polymer/peptide either separates from the underlying NP or adopts an α helix conformation. In either case, uptake of the NP into the tumor cell is increased. Third, the pH-sensitive coating may facilitate disrupting the nanoparticle and releasing the chemotherapeutic drug under acidic conditions. While some coatings only improve targetability, most were designed to accomplish all three objectives. An array of nanoparticles, including zeolith particles [96], block polymeric particles [167], liposomes [168], carbon dots [165], mesoporous silicon particles [169,170], and polypyrrole nanotubes [171] have been coated with pH-sensitive polymers.

The zeolith nanoparticles developed by Tran and Lee could have been placed elsewhere [96]. pH-sensitive coordination and ionic bonds were important in releasing Dox from the zeolith nanoparticles. Nevertheless, we placed the zeolith imidazole framework (ZIF) nanoparticles here because release of the polymer coating was the first step in destabilization of the nanoparticle and release of Dox. They incorporated Dox inside the ZIF that was coated with polyacrylic acid (PAA), functioning as a gatekeeper to prevent Dox leakage from the nanoparticle [96]. The rhombic dodecahedron-shaped ZIF nanoparticles were formed by mixing zinc with 2-methylhistidine. After the negatively charged PAA interacted with the positive ZIF surface, the ZP potential was altered from +37 to −17. While 25% of the Dox was released slowly at pH 7.4 in 100-h, about 85% of the Dox was released at pH 4.0. The pH-dependent release of Dox from the zeolith structure occurred through four stages, including protonation and release of PAA from the nanoparticle, break-up of zinc-imidazole bonds with disassembly of the nanoparticle, and enhanced solubility of the Dox (Table 3). Although the ingenuity in formulating this particle is commendable, the intricacy of the nanoparticle and the potential toxicity of the released zinc may hinder its clinical application. As with other negatively charged polymer coatings, PAA could readily be applied to other positively charged nanoparticles.

Jin et al. also developed a complex multistage-coated nanoparticle to deliver anti-tumor drugs. They incorporated a siRNA, which targeted survivin, and PTX into a PEI-PLA diblock nanoparticle to target lung cancer [167]. While PTX was retained in the hydrophobic PLA core, the siRNA interacted with PEI on the surface. To the surface of the cationic PEI-PLA-siRNA nanoparticle, a negatively charged PEG-poly-L-aspartate (PAsp) diblock polymer was added, which reduced the surface charge from about +28 to +4. The acidic pH of the endosomes should facilitate the release of PAsp-PEG as well as the release of siRNA and PTX from the nanoparticle. Consistent with PAsp release from the nanoparticle at an acidic pH, the ZP became more positive. Furthermore, the release of PTX from the nanoparticle was pH-dependent. About 86% of PTX was released at pH 5.5, whereas 55% of the drug was released at pH 7.4 after 72 h. When injected intravenously into mice, the nanoparticle carrying the siRNA and PTX synergistically reduced the size of 4T1 tumors.

One recently developed nanocarrier that has garnered significant attention is the carbon dot (CD) due to its fluorescence, water solubility, and accessible surface for modification. In one study, both redox- and pH-dependent mechanisms were important in drug release from a carbon dot coated with negatively charged polymers [165]. With the polymer coating, the carbon dots significantly increased in size from less than 10 nm to about 150 nm. The negatively charged coating polymer was released in a mildly acid environment, enabling the positively charged CD to interact with the cancer cell’s surface. The conversion of the negative to the positive polymeric coating was based on the release of the pH-sensitive dimethyl maleic acid (Figure 7B). Masking the charge with dimethyl maleic acid has been used previously with the endosomal lysis melittin peptide [172]. In addition to the pH-sensitive polymer, a redox-sensitive prodrug of cis-platinum was covalently bonded to the CD surface. In contrast to pH 7.4, the cis-platinum-loaded carbon dots at pH 6.8 demonstrated marked toxicity to cancer cells in vitro. Importantly, the cis-platinum-loaded carbon dots modified with the pH-sensitive polymers markedly reduced the tumor xenograft size more than those modified with pH-insensitive polymers [165]. Although carbon dots (CD) have attracted attention due to their bioimaging and drug delivery potential, there are still widespread concerns about their use in humans [173]. While more research is required to determine their toxicity, the coating polymer may have applications with other nanoparticles.

Since problems have occurred with the release of Dox from the FDA-approved liposomal Doxil^®^ product [53], development of liposomal products with improved release of Dox has continued. One such effort led by Miyazaki et al. has been to coat liposomes with several pH-sensitive polymers, such as modified lectins, dextran, and hyaluronic acids (HA) [168,174]. With different modifications of hyaluronic acid, the pKa of the carboxyl groups ranged from 5 to 6.7. Consequently, the distinct pKa ensured that protonation of carboxyl groups would cover the pH range of the tumor matrix and early to late endosomes. Upon acidification, protonation of the carboxyl groups of polymer destabilized the liposomes releasing the entrapped contents. Compared to the other modified HA polymers, the Chex50-HA polymer with a high pKa (between 6.4 and 6.7) and hydrophobicity showed the best results (Figure 4) [168]. There was nearly a 100% release of the fluorescent pyramine from the coated liposomes at pH values of 4.5 within 30 min. In contrast, there was less than 10% of the pyramine release in 30 min. Whether the release profile of pyramine correlates with Dox release is unclear. Moreover, in addition to HA that targeted the CD44 receptor on the malignant cell’s surface, the cyclohexyl groups of Chex50-HA, through hydrophobic interactions, enhanced cellular binding and entry of the modified liposomes. Consistent with entry and the rapid drug release, the Chex50-HA coated liposomes loaded with Dox had greater cytotoxicity toward malignant cells than other polymer-coated liposomes. Unlike PAA and dimethylmaleamide-coating polymers which are not selective, the Chex50-HA polymers are best suited for liposomes due to their destabilizing function.

In addition to polymer-coated particles, a peptide-coated nanoparticle that is pH-responsive shows great promise. For example, Palanikumar et al. determined that Dox-containing nanoparticles coated with a pH-responsive tumor peptide markedly enhanced tumor delivery and antitumor efficacy [27]. The nanoparticles consisted of a polylactic-co-glycolic acid core containing a derivative of Dox (Dox-triphenylphosphonium, TPP), a disulfide crosslinked bovine serum albumin external shell, and an acidity-triggered rational membrane (ATRAM) peptide conjugated to the outer shell. Whereas the ATRAM peptide has a disordered structure at neutral pH, it formed an alpha helix in mildly acid environments (pH 6.5), enabling its insertion into the cellular membrane tumor cell [175,176]. The four glutamic acids within the ATRAM peptide (GLAGLAGLLGL**E**GLL-GLPLGLL**E**GLWLGL**E**L**E**GN) were responsible for the pH-dependent structural conformation.

Interestingly, the authors provide data suggesting that the ATRAM-coated nanoparticles entered the cell through both clathrin-mediated endocytosis and translocation. In addition to increasing cellular uptake, the ATRAM peptide markedly increased the delivery and efficacy of the nanoparticle for tumors in an in vivo model. Moreover, the half-life of the ATRAM-label NP was about 7 h compared to 30 min for free Dox. While the group treated with the Dox-TPP loaded nanoparticles without ATRAM reduced tumor size by 60%, the group treated with nanoparticles coated with ATRAM had tumors that regressed in size [27]. Still, several synthetic steps to form this particle may limit upscaling this product though the ATRAM peptide could readily be attached to other drug-loaded particles. Notably, this was the only drug-loaded NP in this review in which both pharmacokinetics and biodistribution were done. Separate from the nanoparticle, the ATRAM peptide has a prolonged circulatory time of greater than 4 h, probably because of its affinity to albumin. This raises the possibility of whether low molecular weight ATRAM-chemotherapy conjugates would have greater penetration and antitumor efficacy than the larger particles in human cancer.

## 7. Tumor-Penetrating Peptides and Nanoparticles as Potential Chemotherapeutic Carriers

pH-dependent polymers and nanoparticles that have not incorporated or attached chemotherapy agents may still have antitumor properties [44,45,177,178]. These nanoparticles, which become progressively charged as the pH is lowered, may interact with the negatively charged surface of tumor cells, enabling deeper tumor penetration. It would not be surprising that several nanoparticles in this review share this deeper penetration property, but few studies have explored this avenue [123]. In future studies, the following polymers or nanoparticles could readily incorporate chemotherapy or have the chemotherapy administered separately.

For example, Chang et al. synthesized several histidine-leucine peptides with different patterns. One peptide showed antitumor activity with marked apoptosis that was pH dependent. At pH 6.0, the L9H5.1 peptide (40 μM) showed significant toxicity compared to a pH of 7.4 [177]. The enhanced toxicity of the peptides was generally associated with higher helical content. Drug conjugates with the peptide alone or the incorporation of the high leucine content peptide into a nanoparticle are certainly possible.

In addition to peptides, Fan et al. showed that pH-dependent nanoparticles could disrupt the stroma, which can make up 90% of pancreatic tumors [45]. Nanoparticles with random amphipathic copolymers with a pKa of 6.8 effectively disrupted the stroma, but those with a pKa of 6.3 did not. Interestingly, nanoparticles that differed in their pKa were made of co-polymers with similar composition but with different molecular weights (i.e., equivalent amounts of hexylmethacrylate, dimethylaminoethylmethacrylate, and methacrylic acid). At a pH of 6.8, approximating the pHe of tumors, the effective nanoparticle with higher pKa copolymers had a greater positive surface charge, allowing its interaction with membranes of fibroblasts and pancreatic cancer cells in 2-D and 3-D in vitro models. At a pH of 7.4, this co-polymeric nanoparticle did not affect either cell. Whereas the in vivo model only used nanoparticles comprising copolymers to inhibit tumor xenografts, future approaches may use these stroma-disrupting nanoparticles together with gemcitabine or other chemotherapy agents. These investigators also determined that the same pH-disrupting co-polymer which formed nanoparticles inhibited the growth of mouse breast cancer 4T1 cells in vitro and in vivo [44]. Despite the promising results, the partially protonated copolymers (pKa~6.8) may not be sufficiently hydrophobic to incorporate chemotherapy agents stably at pH 7.4.

Similar to the PBAE nanoparticle, Zhang et al. have developed pH-sensitive nanoparticles that depend on polymer degradation to release hydrophobic drugs [178]. They demonstrated that amine-containing polyglyoxylamides rapidly depolymerized end-to-end (“self-immolative”) when exposed to pH 6.0. Furthermore, the pH-sensitive end-caps on these star-shaped polymers significantly affected the degradation rate. Whereas the dimethoxytrityl (DMT) end-cap resulted in rapid degradation of the polymer at acidic pHs, the pH-insensitive carboxybenzyl end cap showed little to no degradation. Similarly, nanoparticles comprising these polymers and DMT end-caps showed similar degradation properties. In addition to the self-immolative properties, the authors suggest that charge–charge repulsion between the polymers had a role in their depolymerization and degradation of the nanoparticles under mildly acidic conditions [178,179]. These pH-dependent polymers are early in their development but have promise as nanoparticle carriers of chemotherapeutic drugs.

## 8. Conclusions

Several ingeniously designed yet incompletely developed nanoparticles are discussed in this review. Some of these nanoparticles have only had their biophysical properties characterized without in vitro or in vivo studies [96,104] (Table 1, Table 2 and Table 3). Other nanoparticles demonstrated promise regarding their stability, pH responsiveness, and in vitro cytotoxicity, but in vivo studies were not initiated [118,121,163]. Still, others showed marked antitumor activity, but their stability at physiological pH may be problematic, and further stabilization of these NPs may be required [116].

Of the 28 pH-disrupting nanoparticles in this review, six examined the in vivo biodistribution of drug-loaded nanoparticles [27,125,127,128,129,167]. These biodistribution studies showed marked tumor inhibition, with two demonstrating tumor regression. Notably, due to the lack of in vivo studies, biodistribution was not done on the nanoparticles incorporating only the chemotherapeutic agent. In contrast, biodistribution experiments were carried out in four of the six drug and nucleic acid combinations. Moreover, pharmacokinetics were completed in two studies [27,130], while pharmacokinetic and biodistribution were just done in one study [27]. Consistent with these results, a limited number of pH-dependent polymeric NP studies have studied the release of the drug during the transit to the tumor [88]. Although potential methods exist to stabilize the nanoparticle-drug interactions, such as cross-linking polymers and enhancing the hydrophobicity of the carrier or the drug, modifications of the NP to increase the half-life cannot be done until baseline studies are completed. To transition the more promising preclinical investigations to clinical trials, further studies will require orthotopic tumor models, long-term stability and lyophilization-reconstitution assays, safety and toxicity profiles beyond the preliminary results, and manufacturing feasibility.

Moreover, to justify further development, there were suggestive studies which showed that pH-sensitive coating particles had markedly more antitumor efficacy than non-coating pH-insensitive particles [27,167,168]. Limited yet more compelling evidence showed superior efficacy of well-control pH-dependent particles compared to pH-independent particles [81,114,119,162,165]. More specifically, direct comparisons between these two groups ranged from the type of bonds to coating polymers. In contrast to the efficacy demonstrated by the pH-sensitive approaches, we did not find studies that showed similar or better efficacy with the pH-independent strategies.

Most of the obstacles for pH nanoparticles are not unique and can be readily applied to both pH-dependent and -independent studies. Moreover, many mechanisms/properties that may enhance EPR (or EPR-like) and tumor penetration for pH-independent particles will also improve the efficacy of pH-dependent particles. Although pH-dependent NPs accumulate more in tumors, this approach is still very dependent on EPR. Even alternative pH-dependent strategies such as immunotherapy or phototherapy, which amplify the tumor efficacy of the drug, will likely be limited by EPR [28,180]. Though a few early approaches enhanced the EPR effect [4,181,182], nanoparticles’ accumulation in tumors was relatively modest with about 30% greater accumulation than by normal tissues [4]. While increases in EPR have been modest, a great deal of research is ongoing to enhance EPR or tumor entry of particles, including transcytosis, induction of nitric oxide with NPs, and hitchhiking on tumor-associated macrophages [183,184,185,186]. Of these strategies, we think that transcytosis will have the most significant immediate impact since the mechanism of entry can be independent as well as supplement EPR [25,184]. We do not think that significant advances in the clinic will happen with either pH-dependent or -independent strategies until tumor entry is improved [187].

Although many shared obstacles exist with pH-independent therapies, at least three differences will require further investigation to enhance pH-dependent strategies. First, to varying degrees, the pH-dependent covalent bonds demonstrate drug released at pH 7.4 in transit to the tumor. In contrast, pH-insensitive covalent bonds are more stable but as we have discussed, this type of bond may be limited by inadequate release in the tumor. By affecting hydrophobicity, progress has been made to stabilize pH-sensitive linkages and NP [88]. As discussed in this review, several pH-disrupting NPs with pH linkages in hydrophobic environments or NPs that co-deliver siRNA and drugs were stable in vitro, but release studies are required to confirm their stability in the circulatory system [128,162]. Second, once entry occurs into the tumor, the NP encounters a mildly acidic pH in the tumor matrix and progressively lower pH in the tumor endosomes, enabling a range of intermediate to complete drug release. While the heterogeneity of extracellular tumor pH has been measured and mapped in selected solid tumors [52], no studies have calibrated the drug’s pH release to specific tumor regions. However, advances have been made in developing pH-responsive nanoparticles that release chemotherapeutic agents at several distinct pHs between 6.4 and 7.2 [120]. Still, the loop to the location in the tumor has not been closed. Third, although there is evidence of differences between the endosomes of the tumor and normal tissues, this has been insufficiently investigated. Unfortunately, there are no imaging methods to measure the pH of tumor (or normal tissue) endosomes in vivo. As pH-sensitive nanoparticles improve, specific knowledge about extracellular tumor pH and endosomal pH will become increasingly important. Without understanding the heterogeneity of the tumor pH of the extracellular matrix and endosomes, the full potential of pH-sensitive nanoparticles and strategies will not be realized.

Many pH-sensitive NPs demonstrated in vivo tumor inhibition or shrinkage. While significant advances are occurring in copolymer synthesis in universities and manufacturing facilities, we think some of these polymers and NPs may currently be too complex to advance beyond clinical trials and have widespread use. These include many graft copolymers, multilayer nanoparticles, and charge reversal particles dependent on releasing pH-sensitive chemotherapeutic and other hydrophobic agents. Seemingly more complicated than NPs delivering single agents, some NPs that co-delivered siRNA and chemotherapeutic drugs demonstrated exceptional in vitro stability and striking antitumor efficacy. These preclinical studies should be confirmed and extended to clinical trials. Although aspects of pH strategies, including pH-linkages and coating peptides/polymers, will find the most immediate application, technological advances for pH-disrupting polymeric NPs will lead to their having a major role in treating patients with solid tumors.

## Figures and Tables

**Table 1:** Charge-charge repulsion with release of chemotherapy and nucleic acids.

Polymer	Drug	Cell Lines (In Vitro, In Vivo)	Comments	Reference
Release of Chemotherapeutic agents				
PBAE ^1^/Pluronic F-108	PTX	NIH3T3 (+, −) ^2^	PBAE polymers stabilized by Pluronic F-108. NPs of about 110 nm completely dissolved at pH 6.5. The pH-sensitive particles loaded with PTX inhibited cells more than pH-insensitive particles.	Lynn et al., 2000 [114]
D-α-tocopheryl-PEG-PBAE	DTX	A2780/A2780-T (+, −)	D-α-tocopheryl inhibits MDR transporter. Diblock polymer formed a NP (size, ~260 nm; ZP, −26 mV). Particle solution became translucent at pH 6.4. Nearly 50% of the drug was released at pH 7.4 in 48 h. Drug-loaded particles inhibited sensitive (IC_50_-0.27 vs. 3.95 μg/mL) and insensitive cells (IC_50_-0.82 vs. 17.7) than the free drug.	Zhao et al., 2013 [116]
mPEG-PBAE-PLA	Dox	−, −	Micelles were formed by triblock polymer (size, 150 nm, ZP, 9 mV). About 20% and 96% of the drug were released from particles at pH 5 after 48 h. Translucency of solution not observed at lower pHs.	Yang et al., 2017 [104]
mPEG—b-PDEAEMA-PMMA/PDEAEMA-b-PMMA	Dox	HepG2 (+, −)	Mixed micelles (~86 nm in size, ZP, ~9.6) formed by diblock and triblock polymers. pH-dependent release of Dox with 20% and 80% released at pH 7.4 and 5.0, respectively, in 60 h. Micelles had modestly less cytotoxicity toward cells than free Dox except at high concentrations. Dox loading content was 24%.	Chen et al., 2017 [118]
PCL-b-PDEAEMA-b-PPEGMA	PTX	NIH3T3 (+, −)	Modest pH-dependent increase in size and release of PTX at pH 5.0. pH-dependent micelles showed modestly greater cytotoxicity toward cells compared to pH-independent micelles.	Feng et al., 2019 [119]
PEG-Dox, PDPAEMA, H4R4	Dox	HeLa (+, −)	Low release of Dox at pH 7.4, yet significant release (90%) of the drug at pH of 5.5 (24 h). H4R4 had no role in drug release but likely enhanced endosomal lysis. NP had markedly improved efficacy toward HeLa cells vs. free drug.	Liang et al., 2015 [103]
P(DEAEMA-r-DPAEMA)	Calcein	NIH/3T3 (+, −)	Mixed micelles between 130 to 160 nm had varied pKa based on the ratios of DEAEMA and DPAEMA incorporated into polymer. Calcein release assay determined endosomal leakage.	Kongkatigumjorn et al., 2018 [120]
PDMAEMA-PDMS (AB5)	Dox	HeLa (+, −)	Empty micelles of diblock polymer in which PDMAEMA had 5 monomeric units were less toxic than those with 13 units. Marked release of Dox at pH 5.5 compared to pH 7.4. At low concentrations, free Dox-inhibited cells more than Dox-loaded micelles.	Car et al., 2014 [121]
Polyiatronic acid-g-FA-PEG-g-PLH	Dox	HeLa (+, −)	Stable micelle at pH 7.4 that showed graded pH release of Dox at pH 7 and less. Greater than 90% of Dox is released at pH 5.0 (24 h). pH-dependent charge surface reversal. Folate-targeted micelle had greater cytotoxicity for HeLa cells compared to free Dox.	Sun et al., 2015 [122]
Star-shaped 5-armed PLGA-His	DTX/ Disulfiram	MCF-7 (+, −)	Marked size increase in micelles at pH 6.8 vs. pH 7.4. Consistent with size increase, micelles released most of the two drugs at pH 6.8. Additionally, the pH-dependent micelles showed increased penetration into MCF-7 spheroid.	Swetha et al., 2021 [123]
Release of Chemotherapy and Nucleic Acids				
PEI-ss-PCL-ss- PEI	Dox/P53-plasmid	HepG2 (+, −)	Dual pH- and redox-responsive NP. Triblock polymers formed a NP with plasmid and Dox of about 168 nm. Modest increase in Dox release in presence of DTT, but pH-responsiveness not done. Significant increase in apoptosis with the combination of Dox and p53 plasmid than either agent alone	Davoodi et al., 2016 [124]
Succinyl chitosan-g-polylysine-palmitic acid	Dox/siPGP	HepG2 (+, +)	pH-responsive micelles (size, ZP) made of graft copolymers. No Dox release studies done. In resistant cells, Dox/siPGP micelles were more cytotoxic (about 3 to 4-fold) than Dox-loaded micelles or free Dox. In vivo, Dox/siPGP micelles reduced tumor size by about 50% more than Dox-alone micelles. Biodistribution study showed tumor specificity of micelle. siPGP reduced resistant PGP levels in tumors in treated mice.	Zhang et al., 2016 [125]
TPGS/poloxamer-PEI conjugate	PTX/ shTw plasmid	4T1 (+, +)	pH-responsive NP in charge, size, and release of Dox and shTw. Charge reversal of NP as pH was lowered. TPGS was necessary for stabilization. PTX-loaded and shTw-loaded particles reduced tumor size and lung metastasis significantly more than PTX-loaded particles in vivo. A biodistribution study showed tumor specificity of NP.	Shen et al., 2012, 2013 [126,127]
PEG-b-PAsp(AED)-b-PDPAEMA	Dox/siBCL-2	SKOV-3 (+, +)	Dual pH- and redox-dependent NP. While 80% of Dox was released at pH 5, 90% was released at pH 5 and with DTT. Synergistic antitumor efficacy in vivo and prolonged survival observed with Dox- and siBCL-2-loaded NP. Biodistribution study showed tumor specificity of NP	Chen et al., 2014 [128]
PEG-b-PLA-PLH-ss-OEI	Dox/siPGP	MCF7/MDR-ADR (+, +)	Dual redox- and pH-dependent polyplex. Polyplex (size, ~120 nm, ZP, +25 mV at pH 7.4) also showed significant levels of cytotoxicity in MCF-7/ADR cells and marked synergistic tumor size suppression in mice. Additionally, biodistribution studies demonstrated tumor specificity of polyplex.	Gao et al., 2019 [129]
PEG-PEI/PEI-PCL	Methotrexate/ siNotch1	Raw264.7 (+, − ^3^)	Non-tumor model in which polymeric NP (~160 nm in size) with a prolonged half-life in blood (~6 h). No pH-dependent studies done. Drug- and siRNA-loaded nanoparticles showed marked reduction in inflammation compared to methotrexate in an in vivo arthritic model.	Zhao and Zhang, 2018 [130]

^1^ Abbreviations. PBAE, poly(β-amino ester); Pluronic F-108, a triblock polymeric stabilizer comprising polyethylene glycol (PEG)-polypropylene (PPO)-PEG; PTX, paclitaxel; NP, nanoparticle; DTX, docetaxel; A2780-T, a drug-resistant ovarian cancer cell line; MDR-multidrug resistant transporter; ZP, zeta potential (mV); PLA, polylactic acid; mPEG, methoxy-polyethylene glycol; Dox, doxorubicin; mPEG-b-PDEAEMA-PMMA, a triblock copolymer, mPEG--poly(2-(diethylamino)ethyl methacrylate-poly-(methylmethacrylate); PCL-b-PDEAEMA-b-PPEGMA, poly(ε-caprolactone)-b-PDEAEMA-(poly(poly(ethylene glycol) methyl ether methacrylate; PTX, paclitaxel; PEG-Dox, a conjugate; PDPAEMA, poly(2-(diisopropyl amino)ethyl methacrylate); H4R4, a peptide comprising the sequence, HHHH-RRRR; P(DEAEMA-r-DPAEMA), a copolymer containing different ratios of (diethylamino)ethyl MA and (diispropylamino)ethyl MA; PDMAEMA-PDMS, poly(2-(dimethylamino)ethyl methacrylate-polydimethylsiloxane; polyiatronic acid-g-FA-PEG-g-PLH, polyiatronic acid-g-folic acid-PEG-g-polyhistidine; PEI-ss-PCL-ss-PEI, a triblock polymer with reducible disulfide (-ss-) bonds; siPGP, a siRNA targeting the PGP transporter; TPGS, D-α-tocopheryl-PEG; MCF7-ADR- MCF7 cell line demonstrating Adriamycin resistance; shTw, a plasmid expressing shRNA which targets the Twist transcription factor; PEG-b-PAsp(AED)-b-PDPAEMA, PEG-b-poly(N -(2,2′-dithiobis(ethylamine)) aspartamide)-b-PDPAEMA; siBCL-2, a siRNA that targets BCL-2; OEI, oligoethylenimine; siNotch1, siRNA targeting Notch-1; PEG-PEI, PEG-polyethylenimine; PEI-PCL, polyethylenimine-PCL. ^2^ + or − indicate whether in vitro or in vivo experiments were done with cells. ^3^ In vivo arthritis model induced in Sprague Dawley rats.

**Table 2:** Nanoparticle disassembly linked to pH-sensitive bonds.

Polymer	Drug/Payload	pH-Sensitive Bond	Cell Lines ^1^ (In Vitro, In Vivo)	Comments	Reference
Dextran	Fluorescein-labeled Dextran (FITC) ^2^	Acetal	RAW macrophages (+, −)	Acetal groups conjugated to hydroxyl groups increased hydrophobicity. The average size of the microsphere was about 240 nm. The release half-life for FITC-dextran at pH 7.4 and 5.0 was about 15 days and 10 h, respectively. Also incorporated ovalbumin stimulated immune response.	Bachelder et al., 2008 [159]
PLLA backbone with interspersed acetal groups	Dox	Acetal	4T1 (+,+)	Acetalized-PLLA microspheres with sizes ranging from 2 to 15 μm. About 30 and 70% of Dox release at 7.4 and 5.0, respectively. 12 days after the 4th intratumoral injection, subcutaneous tumors were inhibited by about 80%.	Li et al., 2018 [160]
Star polymer comprising DMAEMA co-MAEBA-co-DTDMA	Dox	Imine	HeLa, HepG2 (+, −)	Complex polymer synthesis with optimal micelle size of 170 nm. Two different imine interactions with Dox. Dox release at pH 7.4 less than 5% in 48 h, whereas 60% release at pH 5.0 and DTT 10 mM. Targeted Dox-loaded micelles less effective than free Dox except at high Dox concentrations.	Qiu et al., 2015 [158]
PMBC-Polylysine	Dox	Imine	4T1 (+,+)	ε-amino group of lysine formed imine bonds with 4-CB or Dox. Micelles demonstrated pH-dependent charge reversal, size increase, and Dox release. In vivo Dox-loaded micelle inhibited tumors in mice more than free Dox.	Ma et al., 2018 [157]
PEG-b-(PCL-co-DCL)	Dox	β-carboxylic amide	HepG2 (+, −)	Release of pH-sensitive β-carboxylic acid resulted in negative to positive charge reversal micelle. Very pH-responsive micelle. About 10% and 90% of Dox were released at pH 7.4 and 5.3, respectively. Notably, micelles inhibited cells more effectively than pH non-responsive particles.	Deng et al., 2014 [162]
PEG-PAA	PTX, Dox	Acetal	A549 (+, −)	With pH release of PTX from PAA polymer, the negatively charged PAA micelle was disassembled. High loading capacity of 43% with PTX. In contrast to sensitive cells, micelles inhibited PTX-resistant cells significantly more than free PTX. Micelles were stable for months at 4 °C. Additionally, pH-dependent release of Dox was shown.	Gu et al., 2014 [163]
Iodoacetate-modified Keratin	Dox	Hydrazone	A549 (+ in vitro; H22, + in vivo)	Keratin-Dox NP formed by desolvation with size of 250 nm. About 60% of Dox released in 48 h at pH 5, while less than 5% released from particles in 11 days at pH 7.4. Negative to positive charge reversal as pH decreased. Dox-loaded NP inhibited H22 tumors in vivo more than free Dox.	Liu et al., 2019 [164]

^1^ + or − indicate whether in vitro or in vivo experiments were done. ^2^ Abbreviations. FITC, fluorescein isothiocyanate; PLLA, poly(L-lactic acid); Dox, doxorubicin; DMAEMA-co-MAEBA-DTDMA, a star polymer comprising dimethylamino)ethyl methacrylate-co- methacryloxyethoxy)-benzaldehyde)-co- 2,2′-dithiodiethoxyl dimethacrylate; PMBC-polylysine, 2-methacryloyloxy-ethyl phosphorylcholine-polylysine; PEG-b-(PCL-co-DCL), a triblock copolymer, polyethylene glycol-b-(PCL-co-γ-dimethyl maleamidic acid-ε-caprolactone; PEG-PAA; PEG-poly(acrylic) acid.

**Table 3:** pH release of coating polymers from NPs.

Coating Polymer	Nanoparticle	Drug	Cell Lines (In Vitro, In Vivo) ^1^	Comments	Reference
PAA	Zeolith imidazole NP	Dox	ND	Rhombic dodecahedron ZIF was formed by zinc and 2-methylhistidine. PAA-coated positively charged ZIF with size of about 170 nm. ^2^ Several components of NP (coating, zinc-imidazole, Dox) were pH-dependent. 25% and 85% of Dox released from NP at pH 7.4 and 5.0, respectively, in 100 h.	Tran and Lee, 2021 [96]
PAsp-PEG	PEI-PLA NP	PTX, siSurvivin	4T1 (+, −); A549 (+,+)	Coated NPs of about 82 nm enhanced in vivo tumor efficacy with large tumors regressing and prolonged animal survival compared to non-coated NPs. Improved tumor accumulation of NP with coating polymer (biodistribution)	Jin et al., 2018 [167]
PEG-(PAH/DMMA)	CD	Cis-platinum	A2780 (+, −); HeLa (+, −); U14 (+,+)	Release of pH-sensitive dimethyl maleic acid results in the coating polymer release from CD. After decloaking, the cis-platinum conjugated to CD is sensitive to reducing conditions. A2780 and HeLa cells were more sensitive to cloaked NP at pH 6.8 than 7.4. Treated U14-bearing mice regressed with coated Dox-NPs, and pH-polymer was more effective than pH-independent polymer.	Feng et al., 2016 [165]
Chex50-HA	Liposomes	Dox	HeLa, MCF7, Colon 26, NIH3T3 (+, −)	80% release of Chex50-HA in 10 min from NP at pH 4.5. Uptake of coated NP (size, 141 nm) was significantly greater in cells with high expression of the CD44 receptor (HeLa, Colon 26). Coated Dox-loaded NP showed significantly greater cytotoxicity toward HeLa cells than non-coated NP.	Miyazaki et al., 2018 [168]
ATRAM	Cross-linked BSA-PLGA NP	Dox-TPP	Neuro 2A (+, −); HeLa (+, −); MCF-7 (+, −); 4T1, (+,+)	Dual pH and redox-dependent NP about 100 nm in size. Less than 5% and about 80% of Dox-TPP released from NP at pH 7.4 and 5.0 in 24 h. The half-life of 7 h in blood in vivo with enhanced tumor accumulation of NP. Treatment with ATRAM-coated NPs regressed tumors and was more effective than uncoated NPs.	Palanikumar et al., 2020 [27]

^1^ + or − indicate whether in vitro or in vivo experiments were done. ^2^ Abbreviations. PAA, polyacrylic acid; NP, nanoparticle; ND, not done; ZIF, zinc-imidazole framework; PAsp-PEG, polyaspartate-polyethylene glycol; PEI-PLA, polyethyleneimine-polylactic acid; PEG-(PAH/DMMA), PEG-(poly-(allylamine)/dimethyl maleic acid; CD, carbon dots; Chex50-HA, carboxylhexyl50-hyaluronic acid; ATRAM, acidity-triggered rational membrane peptide; BSA-PLGA-NP, bovine serum albumin-polylactic-co-glycolic acid; Dox-TPP, doxorubicin triphenylphosphonium.

## Data Availability

Not applicable.

## Abbreviations

AB5	polymer comprising polydimethysiloxane (PDMS) and PDMAEMA
ADP	adenosine diphosphate
ATP	adenosine triphosphate
Afc	aminoferrocene
ATRAM	acidity-triggered rational peptide comprising GLAGLAGLLG- LEGLLGLPLGLLEGLWLGLELEGN
4-CB	4-carboxybenzaldehyde
CD	carbon dots
Chex50-HA	hyaluronic acid modified by cyclohexyl groups containing a carboxyl group or alkyl esters
4-CB	4-carboxybenzaldehyde
Co-DCL	co-dimethyl maleamidic acid-ε-caprolactone, a copolymer
DEAE	diethyl aminoethyl (pH sensitive subunit of polymer)
DMAE	dimethyl aminoethyl (pH sensitive subunit of polymer)
DPAE	Diisopropyl aminoethyl (pH sensitive subunit of polymer)
Dox	doxorubicin
Dox-Ma	doxorubicin methacrylamide
EGFR	epidermal growth factor receptor
EPR	enhanced permeability and retention
FA	folic acid
HA	hyaluronic acid
HIF-1α	hypoxia inducible factor-1 alpha
His	histidine
H4R4	histidine-arginine peptide
Lys	lysine
MCT	monocarboxylate transporter
NHE-1-10	sodium hydrogen exchanger-1 to 10
MAEBA	(methacryloxyethoxy)-benzaldehyde, a component of the copolymer
MDR	multidrug resistance
MRI/MRS	magnetic resonance imaging/magnetic resonance spectroscopy
NAD	nicotinamide adenine dinucleotide
NADH	nicotinamide adenine dinucleotide hydrogen
NBC	sodium bicarbonate transporter
OEI	oligoethylenimine
PAA	polyacrylic acid
PAsp	poly-L-aspartic acid
PAsp(AED)	poly(N-(2,2′-dithiobis(ethylamine)) aspartamide)
PBAE	poly(β-amino ester) polymers
PCL	poly(ε-caprolactone)
(m) PEG	(methyl) polyethylene glycol
PDEAEMA	poly(2-(diethylamino)ethyl methacrylate)
PDMAEMA	poly(2-(dimethylamino)ethyl methacrylate
PDPAEMA (PDPA)	poly(2-(diisopropylamino)ethyl methacrylate
PGP	P-glycoprotein transporter
pHe	extracellular pH
PEI	polyethylenimine
PLH	poly-L-histidine
PLLA (PLA)	poly-L(L,D)-lactic acid
PLGA	poly lactic-co-glycolic acid
PMMA	mPEG-b-PDEAEMA
PPO	Pluronic F-108
PPEGMA	poly(polyethylene glycol) methyl ether methacrylate
PDMS	polydimethylsiloxane
PMBC	poly(2-methacryloyloxy-ethyl phosphorylcholine
PTX	paclitaxel
shRNA	short hairpin RNA expressed by plasmid
shTw	short hairpin RNA targeting the Twist transcription factor
siBCL-2	siRNA targeting BCL-2
siPGP	siRNA targeting the P-glycoprotein transporter
siRNA	short interfering RNA
TCA	tricarboxylic acid
TMDP	trimethylene dipiperidine, one potential subunit that is part of PBAE
TPGS	D-α-tocopheryl-PEG
V-ATPase	vacuolar ATPase transporter
ZP	zeta potential, which reflects the surface charge of the particle
